# Crystal Structure and Morphology-Controlled Synthesis of Co_1–*x*_Mn_*x*_P Nanocrystals and Their Composition-Dependent Electrocatalytic Activity for the Hydrogen Evolution Reaction

**DOI:** 10.1021/acsami.5c26069

**Published:** 2026-05-16

**Authors:** Md Kawsar Alam, Danyang Wang, Jordon Baker, Ka Un Lao, Indika U. Arachchige

**Affiliations:** Department of Chemistry, 6889 Virginia Commonwealth University, Richmond, Virginia 23284-2006, United States

**Keywords:** water electrolysis, hydrogen evolution reaction, transition metal phosphides, bimetallic nanostructures, electrocatalysis

## Abstract

An important obstacle to long-term hydrogen sustainability is the lack of efficient and stable non-noble-metal catalysts for hydrogen generation through water electrolysis. Cobalt phosphides have emerged as earth-abundant catalysts for the hydrogen evolution reaction (HER), and its activity can be augmented by admixing synergistic elements to produce heteroatom-doped catalysts. Herein, we report an integrated computational and experimental study leading to the synthesis of Co_1–*x*_Mn_*x*_P nanocrystals (NCs) displaying superior activity and stability for the alkaline HER compared to the benchmark Pt/C catalyst at higher current densities (*j* ≥ −35 mA/cm^2^). Density functional theory calculations predicted that Mn doping modulates the hydrogen adsorption energies (ΔG_H_) of orthorhombic CoP toward thermoneutral values. Accordingly, a series of Co_1–*x*_Mn_*x*_P NCs (x = 0.038–0.169) with control over structure, morphology, and composition was produced via colloidal synthesis. Physical characterization of Co_1–*x*_Mn_*x*_P NCs revealed an orthorhombic structure, pseudospherical morphology, and average diameters of ∼5.7–10.2 nm. The incorporation of Mn caused significant modulation of the electronic structure prompting a decrease in Co­(2p) and P­(2p) binding energies, suggesting an increase in electron density on both surface sites. Among NCs investigated, Co_0.909_Mn_0.091_P composition displayed the highest HER activity with an overpotential (η_–10_) of 136.29 mV at *j* = −10 mA/cm^2^, consistent with composition-dependent ΔG_H_ studies. With a Tafel slope of 65.77 mV/dec, Co_0.909_Mn_0.091_P NCs showed similar kinetics to the Pt/C catalyst (62.31 mV/dec), indicating the Volmer-Heyrovsky HER mechanism. The highest-performing Co_0.909_Mn_0.091_P NCs showed a prominent increase in electrochemically active surface area and significantly lower charge transfer resistance compared to parent CoP NCs. The Co_0.909_Mn_0.091_P NCs showed exceptional stability in alkaline media compared to CoP NCs and commercial Pt/C catalysts. Co_0.923_Mn_0.077_P, Co_0.909_Mn_0.091_P, and Co_0.831_Mn_0.169_P compositions displayed superior HER activity and stability compared to monometallic CoP NCs suggesting that dopant-induced compositional and surface modification is an effective strategy for designing high-efficiency, durable nanostructures for numerous heterogeneous (electro)­catalytic studies.

## Graphical abstract

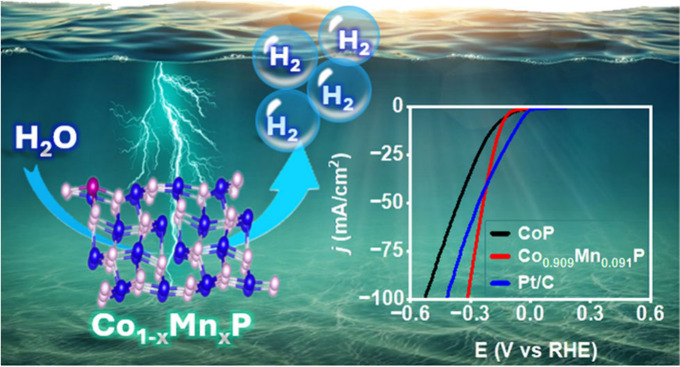

## Introduction

Sustainable energy sources are becoming increasingly important as alternatives to conventional fossil fuels due to the growing energy demand and adverse environmental impacts of fossil fuels.^1–3^ Renewable energy sources, including solar, tidal, and wind, can effectively meet energy demands. However, these sources are unreliable and geographically dependent, which impacts the continuous energy supply.^4,5^ The potential solution involves converting and storing surplus renewable energy as chemical energy, such as hydrogen, for on-demand utilization.^5^ Electrochemical water splitting is a promising approach that can produce hydrogen and oxygen through the efficient breakdown of water by using renewable electricity.^6,7^ This process is environmentally friendly and contributes to the goals of sustainable energy and net-zero carbon emission, as water is the only combustion byproduct produced.^8^ Moreover, hydrogen is an attractive future energy source due to its high gravimetric energy density (143 MJ/kg) in comparison to natural gas (53.6 MJ/kg) and other petrochemicals (42.2–49.6 MJ/kg), as well as high abundance in the universe.^9^ At present, noble metals, such as Pt and RuO_2_ or IrO_2_, are commercially used catalysts for hydrogen (HER) and oxygen (OER) evolution reactions, respectively. However, they are incredibly limited due to scarcity and high cost, which restricts the development of hydrogen as a widespread, sustainable, and renewable energy source.

Recently, there has been a notable interest in developing earth-abundant catalysts, such as transition metal phosphides, borides, chalcogenides, and oxides, for water electrolysis.^10–14^ Among metal phosphides, cobalt phosphides have emerged as promising bifunctional catalysts for both HER and OER.^10,11,15^ CoP has gained notable interest in the HER due to the presence of dual-catalytic sites; the Co site which is responsible for water dissociation and the production of H* intermediates and the phosphorus site, which absorbs H* intermediates and accelerates the desorption of hydrogen gas.^16^ Moreover, efficient electron transfer capability of CoP with minimal ohmic losses to surface sites is expected to promote the HER activity of CoP further.^17^ Additionally, both cobalt and phosphorus can contribute to the HER as the hydride-acceptor and proton-acceptor centers, respectively, and phosphorus can facilitate the formation of metal-hydride intermediates for subsequent hydrogen evolution via electrochemical desorption.^18^ To date, various Co–P phases, including Co_2_P, CoP, CoP_2_, and CoP_3_, have been studied as effective catalysts for water electrolysis.^19–23^ For instance, Zhang et al. synthesized hollow nanocages of CoP, CoP_2_, and CoP_3_ embedded in ZIF-67 for the HER, among them CoP exhibited the highest HER activity with an overpotential (η_–10_) of −116 mV at −10 mA cm^–2^ in 1 M KOH.^19^ Similarly, Hua et al. produced heterostructured CoP/Co_2_P nanosheets that showed notably higher HER activity compared to single-phase CoP and Co_2_P.^23^ Furthermore, Yang et al. synthesized several Co-based catalysts (CoP, CoS, and Co_3_O_4_).^24^ Among these, CoP demonstrated the highest HER activity which has been attributed to its accessible active sites and higher conductivity. Nonetheless, the HER activity of CoP is inferior to the benchmark Pt/C (10%) catalyst because of slower interfacial charge transfer, higher overpotentials, and a larger Tafel slope.^16,25–28^ Similarly, the hydrogen adsorption Gibbs free energy (ΔG_H_) of CoP deviates significantly from the ideal energy range (ΔG_H_ ± 0.1 eV) required to satisfy Sabatier’s principle for optimal HER performance.^24,29^

An effective strategy to enhance the HER activity of cobalt phosphides is the admixing of synergistic metals to produce heteroatom-doped CoP. This process tends to alter catalyst’s properties, including crystal structure,^11^ morphology,^30^ and surface polarization,^10^ leading to improved HER performance.^31,32^ To date, cobalt phosphides doped with Zn,^21^ V,^33^ Fe,^34^ Al,^27^ Mo,^35,36^ Mn,^37–39^ Cr,^28^ and Ru^40^ have been reported for the HER. For instance, Mn-doped CoP nanosheet arrays are reported to show promising HER activity with an overpotential (η_–10_) of 76 mV at −10 mA cm^–2^ in alkaline electrolytes where the performance gain is attributed to thermo-neutral ΔG_H_ achieved upon Mn doping.^37^ Similarly, Mn-CoP nanowire@Mn-CoOOH nanosheet arrays with defect-related P vacancies^39^ have been reported to lower the hydrogen adsorption energies closer to a thermoneutral values, enhancing the HER activity. Zhao et al. reported the synthesis of Mo-doped CoP with lattice distortions and structural defects.^36^ These modifications caused an increase in surface active sites and improved HER activity with η_–10_ of 88.4 mV in alkaline electrolytes. In another study, admixing Al has been shown to decrease the ΔG_H_ of pristine CoP from −0.14 eV to −0.08/–0.02 eV, indicating a notable increase in HER performance.^27^ Here, incorporation of Al has been reported to increase the double-layer capacitance and lower the charge-transfer resistance (R_ct_) of parent CoP. Similarly, Ru-doped CoP nanosheets showed exceptional HER activity in acid and alkali (η_–10_ = 49 and 51 mV, respectively) owing to an increase electron density closer to Fermi level upon incorporation of synergetic elements.^40^ Although a few heteroatom-doped Co–P have been investigated for the HER, the focus has always been on a randomly selected dopant with a fixed dopant composition. Besides dopant identity, dopant concentration also influences the HER activity and stability.^10,11,41^ However, to our knowledge, a systematic composition-dependent HER activity investigation of heteroatom-doped Co–P has not been reported, impeding its development as a promising water splitting catalyst.

Herein, a computational analysis of several synergistic dopants (Cr, Fe, Mo, V, and Mn) was conducted to evaluate their influence on ΔG_H_ on orthorhombic CoP (011) and Co_2_P (201) surfaces. DFT calculations on orthorhombic CoP (011) indicate Mn as an optimal HER dopant that lowers the overall hydrogen adsorption energy, |ΔG_H1_| + |ΔG_H2_| = 0.07 eV, closer to a thermoneutral value. In contrast, heteroatom-doped orthorhombic Co_2_P (201) showed a notably higher |ΔG_H1_| + |ΔG_H2_| value, suggesting a lower HER activity. Accordingly, a series of phase-pure Co_1–*x*_Mn_*x*_P NCs (x = 0.038–0.169) was colloidally synthesized to examine the influence of dopant composition on HER activity, without deconvoluting factors from structure and morphology. Parent CoP NCs displayed η_–10_ = 166.70 mV, showing a notable HER activity consistent with literature.^42,43^ Upon incorporation of Mn, the highest HER activity was observed for Co_0.909_Mn_0.091_P NCs, corresponding to Mn concentration of 5.091%, consistent with composition-dependent ΔG_H_ calculations. The highest performing Co_0.909_Mn_0.091_P composition reached η_–10_, η_–20_, η_–50_, and η_–100_ at 136.29, 156.25, 186.53, 214.91 mV, respectively and outperformed the HER activity of benchmark Pt/C catalyst at higher current (*j* ≥ −35 mA cm^–2^). Moreover, the stability analysis with chronopotentiometry showed a negligible change in η_–10_ for Co_0.909_Mn_0.091_P NCs (Δη_–10_ = 5.83% increase) compared to 16.13 and 54.38% increases observed for parent CoP and benchmark Pt/C catalysts, respectively after 10 h of alkaline HER. This indicates that Mn doping enhances both the HER activity and stability of CoP NCs.

## Experimental Section

### Materials

Oleylamine (OLA, 70%), hexane, and ethanol were purchased from Fisher. Dicobalt octacarbonyl (Co_2_(CO)_8_, 90%), manganese carbonyl (Mn_2_(CO)_10_, 98%), and 1-octadecene (ODE, 90%) were purchased from Sigma-Aldrich. Tri-n-octylphosphine (TOP, 97%) was purchased from Strem Chemicals. Carbon-coated 200 mesh copper grids were purchased from SPI Supplies. Titanium foils (thickness 0.25 mm; 99.7%) and platinum on graphitized carbon (Pt/C, 10% wt.) were purchased from Sigma-Aldrich. Graphite rods (6.15 mm × 102 mm, 99.99995%) were purchased from Alfa Aesar. A Hg/HgO reference electrode filled with 1 M NaOH was purchased from CH Instruments. Pelco colloidal silver paint was purchased from Ted Pella. Henkel Loctite EA-9462 epoxy adhesive was purchased from Ellsworth Adhesives. Chemical-resistant PTFE silver-plated copper wire was purchased from McMaster-Carr. OLA was vacuum-dried at 150 °C for 3 h prior to use. Molecular sieves and CaO were used to dry the ethanol, which was subsequently distilled under nitrogen before use.

### Synthesis of CoP NCs

In a 100.0 mL round-bottom flask, 10.0 mL of ODE and 1.5 mL of OLA were added under nitrogen atmosphere and connected to a Schlenk line. This solution was degassed at 120 °C for 30 min and purged with nitrogen, and the temperature was increased to 200 °C. Then, 1.46 mmol of Co_2_(CO)_8_ (0.5 g) dissolved in 5.0 mL of ODE was injected into the OLA/ODE mixture, and the flask was maintained at 200 °C for 20 min. Afterward, 12.0 mL of TOP (26.88 mmol) was introduced, temperature was increased to 340–350 °C, and the reaction mixture was refluxed for 3 h. Then, the reaction was cooled to room temperature, and CoP NCs were precipitated by adding ethanol and centrifuging for 12 min at 6000 rpm. After that, the precipitate was sonicated in 10 mL of hexane to disperse NCs. A minimum of 3 repetitions of this isolation and purification procedure were performed to purify CoP NCs. Finally, the purified black powder was dried under vacuum.

### Synthesis of Co_1–*x*_Mn_*x*_P Alloy NCs

Co_1–*x*_Mn_*x*_P NCs were synthesized using a modified procedure developed for CoP synthesis. Briefly, 10.0 mL of ODE and 1.5 mL of OLA were added to a 100.0 mL round-bottom flask under nitrogen. This flask was connected to a Schlenk line, the solution was degassed at 120 °C for 30 min, followed by purging with nitrogen, and the temperature was raised to 200 °C. Then, appropriate amounts of Co_2_(CO)_8_ and Mn_2_(CO)_10_ precursors (Table 2, 1.46 mmol total) were combined with 5.0 mL of ODE at 50 °C under nitrogen and injected into the reaction mixture at 200 °C. Next, the flask was maintained at 200 °C for 20 min, and 12.0 mL of TOP was injected. Finally, the reaction temperature was increased to 340–350 °C, and the mixture was refluxed for 3 h. As-synthesized Co_1–*x*_Mn_*x*_P NCs were isolated and purified using hexane and ethanol, similar to CoP NCs.

### Physical Characterization

Powder X-ray diffraction (PXRD) patterns were recorded using an Empyrean Multipurpose X-ray Diffractometer equipped with Cu Kα (λ = 1.5418 Å) radiation, under 45 kV and 40 mA operating conditions. The crystallite size was determined by applying the Scherrer equation to orthorhombic CoP (103) reflection. Transmission electron microscopy (TEM) and scanning TEM (STEM) images, elemental maps, and selected-area electron diffraction (SAED) patterns were acquired using a JEM-F200 Cold FEG electron microscope operating at 200 kV. The NC composition was obtained using a Hitachi Ultra High-Resolution Analytical FE-SEM SU-70, equipped with an in situ energy-dispersive spectroscopy (SEM-EDS) analyzer operating at 15 kV. The atomic percentages of five individual spots per sample were measured and averaged to determine the overall elemental composition. A PHI VersaProbe III Scanning XPS Microprobe was used to record X-ray photoelectron spectra (XPS) of all samples. Prior to XPS, NCs were annealed in a tube furnace under 5% H_2_:Ar at 450 °C for 2 h. The pass energy for the regional scans was 26.00 eV, and the step durations were 100 ms for Co and P and 200 ms for Mn. The number of sweeps was varied according to the signal intensity of each element. MultiPak software was used for spectral deconvolution and analysis. The Fourier transform infrared (FT-IR) spectra were recorded using a Thermo Scientific Nicolet iS50 FTIR instrument. The Horiba LABram HR confocal Raman spectrometer equipped with a 532 nm laser was used to record Raman spectra of all samples. Thermogravimetric analysis (TGA) was carried out under nitrogen atmosphere using a TA Instrument Q5000 analyzer. A constant rate of 50 °C/min was used to heat TGA samples.

### Computational Methods

DFT calculations were carried out using the Vienna Ab-initio Simulation Package (VASP), employing the Projector Augmented Wave (PAW) method with the Perdew–Burke–Ernzerhof (PBE) functional for structural optimization.^44^ Adsorption energies (ΔE) were calculated using the PBE functional with the Grimme D3­(BJ) dispersion correction.^45^ A plane-wave cutoff energy of 300 eV and a Γ-centered 2 × 2 × 1 k-point grid were employed to reduce computational cost, as investigations on our systems showed this setup provides comparable performance to calculations performed with a 520 eV cutoff and a Γ-centered 5 × 5 × 1 k-point grid. The ΔG_H_ on the orthorhombic Co_2_P (201) surface with Co_7_P_4_ termination was modeled using a four-layer slab with a 15 Å vacuum along the *c*-axis, using lattice parameters of a = 6.90 Å, b = 10.10 Å, and c = 21.58 Å. Similarly, for the orthorhombic CoP (011) surface with Co_8_P_8_ termination, a four-layer slab with a 15 Å vacuum was used with cell parameters of a = 6.54 Å, b = 10.12 Å, and c = 20.54 Å. With both models, atoms in the bottom layer were fixed to their bulk positions to accurately represent the bulk-like environment. To identify the optimal dopant, five transition metals (Cr, Fe, Mn, Mo, and V) were investigated at a concentration of 3.13%. The ΔG_H_ values were computed using the equation: ΔG_H_ = ΔE + ΔE_ZPE_ – TΔS. Given the focus on comparative catalytic performance across different dopants, the combined entropic and zero-point energy correction term (ΔE_ZPE_ – TΔS) was approximated as 0.24 eV, following literature precedent.^44^ Thus, ΔG_H_ is simplified to ΔE + 0.24 eV. Additionally, a composition-dependent study was conducted on the Mn-doped orthorhombic CoP (011) surface by substituting two and three surface Co atoms with Mn, corresponding to 6.26 and 9.39% Mn compositions, respectively.

### Fabrication of Working Electrodes

The working electrodes of CoP and Co_1–*x*_Mn_*x*_P NCs and commercial Pt/C (10% wt.) catalysts were fabricated on Ti foil substrates. To begin, rectangular pieces of Ti foil measuring 0.5 cm × 0.4 cm were cut, flattened using a hydraulic press, and sonicated in a mixture of acetone and ethanol (1:1, v-v). These foils were dipped in a solution of 1 M HCl and 30% H_2_O_2_ (1:1, v-v) for 20 min and washed with deionized water. A homogeneous catalyst ink was produced by sonicating 4 mg of NCs in 450 μL of isopropanol, 50 μL of Nafion, and 20 μL of ultrapure water for 2 h in a cold-water bath. Then, 50 μL of ink was drop-cast on the Ti foils and annealed at 450 °C for 2 h under 5% H_2_:Ar to eliminate residual surface ligands. Next, a Ag-plated Cu wire was attached to Ti foils using Ag paint. Once the Ag paint was fully dried, a thoroughly mixed two-part epoxy was applied to cover the Cu wire and Ag paint, keeping only the ink drop-casted region (0.20 cm^2^) accessible for HER. The epoxy was dried for ∼12 h prior to HER studies.

### Electrocatalytic Studies

A CHI 760E model electrochemical workstation was utilized for all electrocatalytic experiments. A typical three-electrode setup was used, consisting of a Hg/HgO (1 M NaOH) reference, a graphite rod counter, and a working electrode fabricated with CoP or Co_1–*x*_Mn_*x*_P NCs or the Pt/C (10%) catalyst. All electrodes were immersed in a nitrogen-saturated 1 M KOH solution to record linear sweep voltammetry (LSV) plots. The potential values were converted from the Hg/HgO reference electrode to the reversible hydrogen electrode (RHE) using the conversion formula: E_RHE_ = E_exp_ + E_Hg/HgO_^0^ + 0.05916 pH. Prior to LSV measurements, the working electrodes were electrochemically cleaned using cyclic voltammetry (CV) sweeps. These CV scans involved sweeping the potential in the nonfaradaic region from 0.1 to 0.2 V at 25, 50, 100, and 200 mV/s. The double-layer capacitance (C_DL_) was calculated from these CVs to determine the electrochemically active surface area (ECSA).^46–49^ For each CV, the first step was to determine the difference in anodic and cathodic current densities at a potential of 0.15 V vs RHE (Δ*j*_0.15 V_). Next, a graph of Δ*j*_0.15 V_/2 vs scan rate was plotted and linearly fitted to calculate the slope, which is equivalent to C_DL_. Finally, ECSA was derived from C_DL_/Cs, where Cs represents the specific capacitance (C_s_ = 40 μF/cm^2^). LSVs were recorded by sweeping the potential from 0.2004 V to −0.8000 V (vs RHE) at a scan rate of 5 mV/s. Electrochemical impedance spectroscopy (EIS) was performed over a frequency range of 10^5^ to 0.1 Hz. Unless otherwise noted, all LSV measurements were *iR*-corrected using the sample-specific solution resistance obtained from EIS. Finally, the electrochemical stability of catalysts was determined using a chronopotentiometry (CP) technique conducted at a *j* = −10 mA/cm^2^ for 10 h in nitrogen-saturated 1 M KOH.

## Results and Discussion

### Computational Investigation of the HER Activity of Co_2_P, CoP, and Mn-Doped CoP Catalysts

To identify the optimal transition metal-doped CoP or Co_2_P compositions for HER, five dopants (Cr, Fe, Mn, Mo, and V) were investigated each at 3.13% concentration. In these calculations, ΔG_H_ was used as the primary descriptor for HER activity, where values closer to zero indicate favorable adsorption and desorption of hydrogen species on the catalyst surface.^50^ Cobalt phosphides exhibit multiple stoichiometries, among which Co_2_P and CoP are the most representative phases. Owing to their distinct Co/P ratios (2:1 for Co_2_P and 1:1 for CoP), both compounds exhibit markedly different electronic structures and hydrogen adsorption energetics. To establish a direct comparison, we first evaluated ΔG_H_ on the highest HER active CoP (011) and Co_2_P (201) surfaces. As shown in Supporting Information, Table S1, orthorhombic Co_2_P intrinsically exhibits higher ΔG_H_ values. Even its best-performing heteroatom-doped variant, V–Co_2_P (ΔG_H1_ = −0.47; ΔG_H2_ = −0.25 eV), remains inferior in HER activity to the least favorable doped CoP system, Fe-CoP (ΔG_H1_ = −0.34; ΔG_H2_ = −0.17 eV). This indicates that Co–P with a surface exposing the 1:1 Co:P atomic ratio generally exhibits superior HER performance. Thus, subsequent computational studies were focused entirely on the orthorhombic CoP system.

The first and second ΔG_H_ values for pristine and heteroatom-doped (Cr, Fe, Mn, Mo, and V) CoP (011) surfaces, along with representative structural configurations, are presented in Figure 1, with numerical results summarized in Table 1. Orthorhombic CoP (011) was selected as a representative low-index surface for our DFT studies because it is widely recognized in literature as a thermodynamically stable and catalytically relevant facet for HER.^51–53^ PXRD patterns of pristine and Mn-doped CoP NCs exhibit a clear and intense (011) diffraction peak, which corresponds to the second-highest intensity reflection in the orthorhombic CoP reference pattern (JCPDS No. 04–003–2072). Various adsorption sites were examined, including Co and P top sites, Co–Co bridges, Co–P bridges, and P–P bridges. In most cases, the Co–Co bridge was identified as the most favorable site for the first hydrogen adsorption, while the Co top site was preferred for the second hydrogen adsorption. However, for Fe- and Mn-doped surfaces, the second hydrogen preferentially adsorbed at the bridge site between Co and dopant. These findings suggest that the role of the dopant exceeds a simple ligand effect. The Mn dopant not only modulates the electronic structure of neighboring atoms but also directly contributes to the formation of new, more favorable coordination environments for H adsorption, consistent with recent literature.^11^ For pristine CoP, the overall ΔG_H_ binding energy, expressed as |ΔG_H1_| + |ΔG_H2_|, is 0.18 eV. Upon substitutional doping of Co sites with synergetic elements, the overall ΔG_H_ generally decreases, with the exception of Fe doping. Specifically, Mn, Cr, Mo, and V dopants yielded overall |ΔG_H_| values of 0.07, 0.09, 0.16, and 0.16 eV, respectively, suggesting an increase in HER performance. In contrast, Fe doping led to excessively strong hydrogen adsorption, impairing desorption, and thereby causing a decrease in HER activity. For Mn, Cr, Mo, and V dopants, the improvement in HER performance arose primarily from a reduction in the binding strength of the second hydrogen, while the first hydrogen adsorption remains only slightly affected. These findings suggest that admixing of transition metals, particularly Mn and Cr, can significantly enhance the HER activity of the CoP (011) surface by modifying the overall ΔG_H_ closer to a thermoneutral value. Given its synthetic feasibility, Mn was identified as the optimal dopant for orthorhombic CoP, and subsequent studies were directed toward systematically investigating the influence of Mn composition on HER activity of Co_1–*x*_Mn_*x*_P NCs.

**Figure 1:**
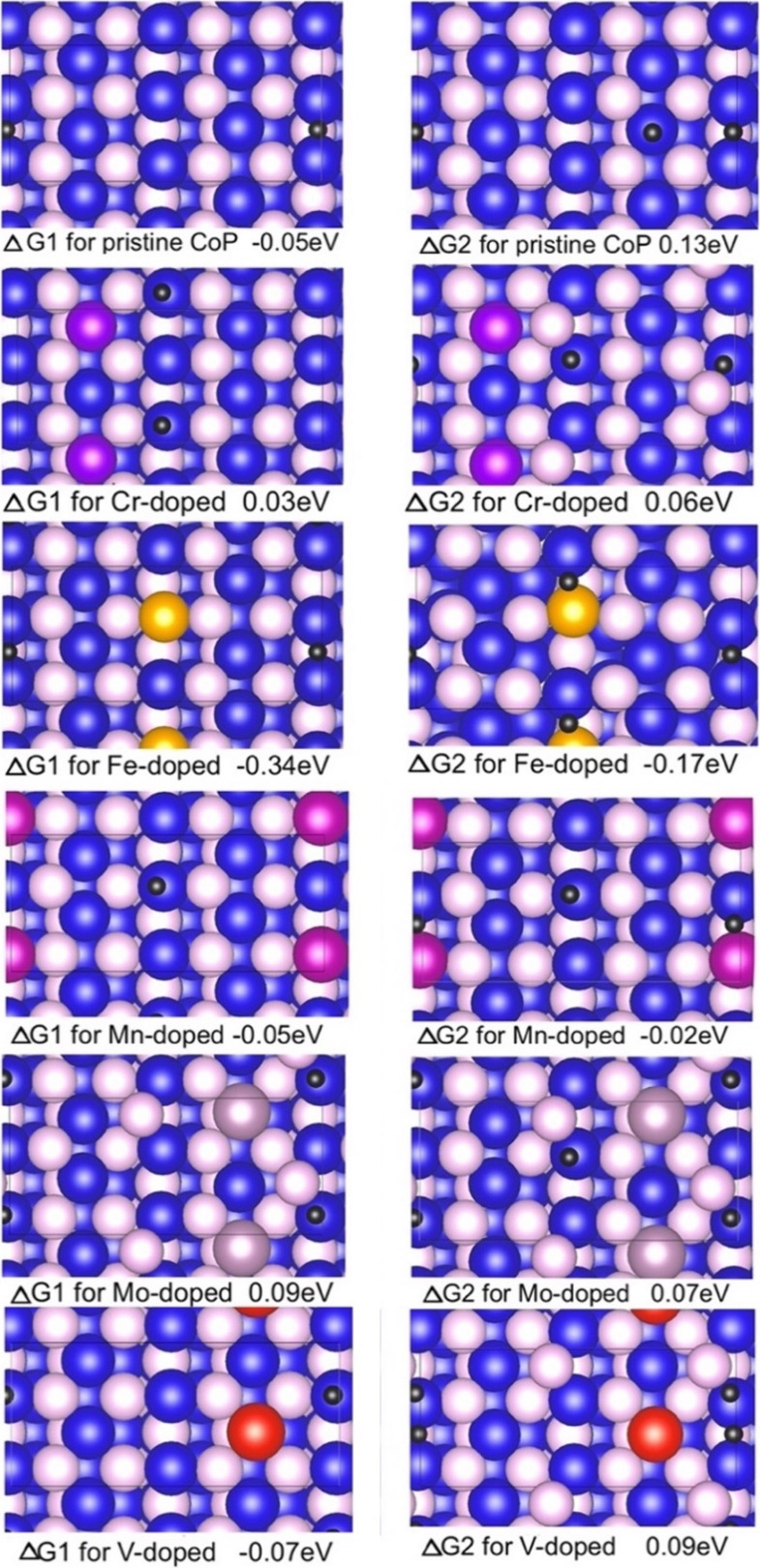
ΔG_H_ values for the first and second hydrogen adsorption on pristine and transition metal-doped (Cr, Fe, Mn, Mo, and V) CoP (011) surfaces with Co_8_P_8_ termination at a dopant concentration of 3.13%. In the structural models, Co atoms are shown in dark blue, P atoms in pink, Cr atoms in purple, Fe atoms in yellow, Mn atoms in magenta, Mo atoms in dark pink, V atoms in red, and H atoms in black color.

**Table 1:** ΔG_H_ Values for the First and Second Hydrogen Adsorption on Pristine and Transition Metal-Doped CoP (011) Surfaces with Co_8_P_8_ Termination at a Dopant Concentration of 3.13%^a^

CoP	ΔG_H1_ (eV)	ΔG_H2_ (eV)	|ΔG_H1_| + |ΔG_H2_| (eV)
Pristine	–0.05	0.13	0.18
Cr	0.03	0.06	0.09
Fe	–0.34	–0.17	0.51
Mo	0.09	0.07	0.16
V	–0.07	0.09	0.16
Mn (3.13%)	–0.05	–0.02	0.07
Mn (6.26%)	0.02	–0.14	0.16
Mn (9.39%)	–0.03	–0.15	0.18

a Additional data obtained for Mn doped systems at 6.25 and 9.39% dopant concentrations are also shown.

Furthermore, a composition-dependent study was conducted to systematically evaluate the influence of Mn doping levels on the HER activity of the CoP (011) surface. When two surface Co atoms were substituted with Mn corresponding to 6.25% Mn composition, the calculated ΔG_H1_ and ΔG_H2_ were = 0.02 and −0.14 eV, respectively (Supporting Information, Figure S1). In this system, the first H atom migrated from the Co atop site to the Co–Mn bridge site, while the second H atom moved from the Co–Mn bridge site to the Mn atop site. Increasing the Mn substitution to three Co sites (9.38% Mn) resulted in ΔG_H1_ = −0.03 eV and ΔG_H2_ = −0.16 eV, with the first H atom remaining at the Co–Mn site and the second H atom absorbed at the Mn atop site. Although ΔG_H_ values at these higher doping levels remain favorable for the HER, the combined absolute binding strengths (|ΔG_H1_| + |ΔG_H2_|) increase progressively, deviating slightly from the thermoneutral condition: 0.07, 0.16, and 0.19 eV for 3.13, 6.26, and 9.39% Mn, respectively (Table 1). This trend suggests that while Mn doping enhances HER activity, excessive doping may overstabilize hydrogen adsorption, thereby hindering desorption and reducing catalytic efficiency. Based on these results, the optimal HER performance is predicted at approximately 3.13% Mn doping, where hydrogen binding energies are balanced to promote efficient catalytic turnover.

### Synthesis and Characterization of Orthorhombic CoP and Co_1–*x*_Mn_*x*_P NCs

Computational studies predicted that Mn-doped CoP outperforms the HER activity of pristine orthorhombic CoP. Accordingly, a series of Co_1–*x*_Mn_*x*_P NCs with control over structure, morphology, and variable compositions was colloidally synthesized to investigate the HER activity in alkaline electrolytes. The synthesis of orthorhombic CoP NCs involves thermal decomposition of the Co_2_(CO)_8_ precursor in ODE, OLA, and TOP surfactant/solvent systems. Here, ODE serves as the solvent, OLA serves as the surfactant, and TOP serves as the phosphorus precursor and a surfactant. During the synthesis, thermal decomposition of Co_2_(CO)_8_ or Co_2_(CO)_8_/Mn_2_(Co)_10_ is expected to produce single element Co or Co–Mn alloy NCs, respectively at low temperature (200 °C).^54–56^ Subsequent phosphidation with alkyl phosphines (TOP) at 350 °C for 3–4 h consistently yielded CoP or Co_1–*x*_Mn_*x*_P NCs with an orthorhombic structure (Scheme 1). Phase-pure CoP NCs can be selectively produced by using a P:Co molar ratio of 18.4. Reducing the temperature to 300 °C prompted the growth of a phase mixture of CoP and Co_2_P NCs. As-synthesized Co_1–*x*_Mn_*x*_P NCs retained the orthorhombic structure of CoP without any impurities; hence, the incorporation of Mn_2_(Co)_10_ did not affect the phase purity of ternary NCs. However, when the molar ratio of Co_2_(CO)_8_:Mn_2_(CO)_10_ is <1.5, a heterogeneous mixture of Mn-doped CoP and Co_2_P NCs was obtained at ∼340 °C. In contrast, Co and Co–Mn alloy NCs that were initially formed from a Co:Mn precursor ratio of ≥1.5 consistently undergo complete phosphidation at ∼340 °C, yielding phase-pure, orthorhombic Co_1–*x*_Mn_*x*_P NCs.^54^

**Scheme 1:**
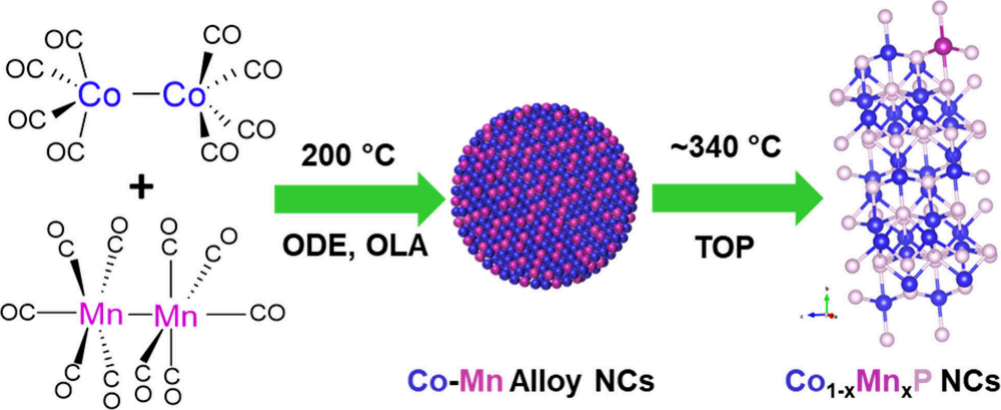
Schematic Representation of the Synthesis of Orthorhombic Co_1–*x*_Mn_*x*_P NCs

The elemental compositions of Mn-doped CoP NCs were examined using SEM/EDS, and the average values are shown in Table 2. The EDS analysis revealed that the concentration of Mn varies from 2.07% to 9.99% in Co_1–*x*_Mn_*x*_P NCs, corresponding to x = 0.038–0.197. The experimental Mn content is lower than the nominal molar ratio of Co_2_(CO)_8_:Mn_2_(CO)_10_ precursors used in the synthesis. Typically, a nominal concentration of 20% Mn_2_(CO)_10_ can produce Co_1–*x*_Mn_*x*_P NCs with an experimental Mn composition of ∼5%. Similarly, experimental Mn compositions of ∼2–3% or 8–10% can be achieved with nominal Mn_2_(CO)_10_ concentrations of 10–15% or 30–40%, respectively. A mixture of orthorhombic Co_2_P, CoP, and orthorhombic MnP nanostructures was obtained when the nominal Mn_2_(CO)_10_ concentration was >40%. Overall, the TOP/Co_2_(CO)_8_ molar ratio of 18.4 and a phosphidation temperature of 340–350 °C are the optimal parameters for synthesizing phase pure, orthorhombic CoP NCs. In contrast, orthorhombic Co_1–*x*_Mn_*x*_P NCs were consistently obtained when a P/total metal molar ratio of 18.4 and a nominal Mn_2_(CO)_10_ concentration of ∼10 to 40% were used at a synthesis temperature of ∼340 °C.

Binary CoP and Mn-doped CoP NCs were analyzed via PXRD to investigate the structure, crystallinity, and phase purity of samples. Figure 2 illustrates the diffraction patterns of selected Co_1–*x*_Mn_*x*_P compositions with x = 0 to 9.99%. Parent CoP NCs exhibit orthorhombic structure, and the diffraction patterns can be indexed to the JCPDS file No. 04–003–2072. Impurity phases, such as orthorhombic Co_2_P, cubic Co, and corresponding oxides, were not detected in any of the samples examined, suggesting that the bimetallic NCs are phase pure. Upon admixing Mn, the orthorhombic structure of CoP was maintained in Co_1–*x*_Mn_*x*_P NCs up to x = 9.99%. Diffraction patterns of Co_1–*x*_Mn_*x*_P NCs show no obvious shifts with increasing dopant concentration, suggesting that Mn neither expands nor contracts the orthorhombic CoP structure. This is not surprising, as Mn and Co are relatively similar in size (Co = 125 pm and Mn = 127 pm); therefore, no changes in the lattice parameter are expected. The crystallite size of each NC composition was calculated by applying the Scherrer formula to (103) reflection. Binary CoP NCs displayed an average crystallite size of ∼9.3 nm, whereas Co_1–*x*_Mn_*x*_P NCs exhibited average sizes from 6.4–9.9 nm for x = 0.038–0.169 compositions (Table 2)­. It is likely that kinetics and microstructural factors, rather than atomic size mismatch, are responsible for the relatively smaller crystallite size observed upon Mn doping. It can be assumed that Mn alters the relative reactivity of precursors that can potentially change the nucleation rate, delaying the growth of ternary NCs.^57^ The crystallite sizes estimated from Scherrer broadening of (103) peak are in agreement with the average particle sizes obtained from TEM imaging. The smaller crystallite sizes inherent to Co_1–*x*_Mn_*x*_P NCs increase the surface area, which could be beneficial for catalytic studies.

**Figure 2:**
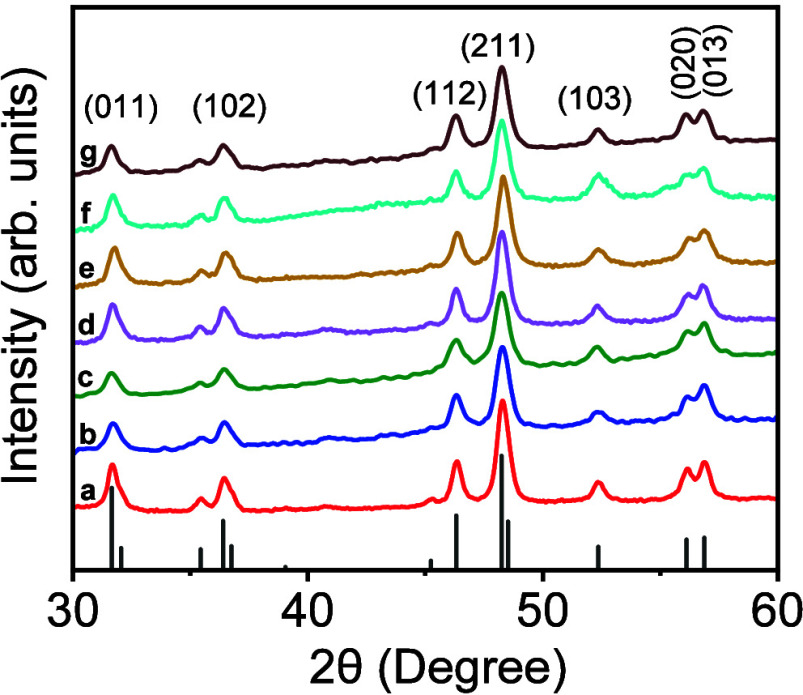
PXRD patterns of CoP and Co_1–*x*_Mn_*x*_P alloy NCs with variable compositions: x = (a) 0, (b) 0.038, (c) 0.053, (d) 0.077, (e) 0.091, (f) 0.130, and (g) 0.169. The ICCD PDF overlay of orthorhombic CoP (JCPDS No. 04–003–2072) is shown as vertical black lines.

**Table 2:** Nominal and Experimental Compositions and Crystallite and Average Particle Sizes of Orthorhombic CoP and Co_1–*x*_Mn_*x*_P NCs Synthesized via Colloidal Synthesis

	Elemental Composition center (EDS)^a^					
Sample Name	Co	Mn	P	Nominal Mn Composition	Crystallite Size, nm (PXRD)^b^	Particle Diameter, nm (TEM)^c^
CoP	53.695	0	46.305	0%	9.3	8.4 ± 1.1
Co_0.962_Mn_0.038_P	52.852	2.068	45.081	10%	8.4	10.2 ± 1.5
Co_0.947_Mn_0.053_P	52.396	2.946	44.658	15%	7.7	9.9 ± 1.8
Co_0.923_Mn_0.077_P	48.478	4.028	47.495	17%	7.3	5.7 ± 0.7
Co_0.909_Mn_0.091_P	50.906	5.092	44.002	20%	9.9	7.4 ± 1.1
Co_0.870_Mn_0.130_P	53.181	7.958	38.862	30%	6.4	5.9 ± 0.9
Co_0.831_Mn_0.169_P	47.901	9.722	42.378	40%	9.5	8.5 ± 1.3

a Elemental compositions were obtained from the SEM/EDS analysis. Each composition was determined by averaging five individual measurements per sample.

b Crystallite sizes were determined by applying the Scherrer equation to the (103) reflection of the corresponding PXRD patterns.

c Average particle diameters were computed from 150 to 180 discrete particles obtained from several HRTEM and LRTEM images.

As-synthesized NCs were annealed at 450 °C for 2 h in 5% H_2_/Ar prior to HER experiments. This annealing step did not alter the structure or composition of CoP and Co_1–*x*_Mn_*x*_P NCs (Supporting Information, Figure S2). However, a modest increase in crystallite size to 9.7–13.7 nm occurred for Co_1–*x*_Mn_*x*_P NCs with x ranging from 0.038 to 0.091.

TEM was used to investigate the morphology and size dispersity of CoP and Co_1–*x*_Mn_*x*_P NCs, and representative micrographs and size histograms are shown in Figure 3 and Supporting Information, Figure S3, respectively. Binary CoP NCs exhibit spherical morphology and an average diameter of 8.4 ± 1.1 nm. At low Mn incorporation (x = ∼2–3%), Co_0.962_Mn_0.038_P and Co_0.947_Mn_0.053_P NCs exhibit slightly larger diameters, which are comparable to parent CoP, suggesting that low Mn incorporation minimally affect the NC growth (Table 2). Conversely, at higher Mn incorporation (∼4–8%), significantly smaller average diameters were obtained for Co_0.923_Mn_0.077_P, Co_0.909_Mn_0.091_P, and Co_0.870_Mn_0.130_P NCs, likely due to inhibition of crystal growth by the Mn precursor.^58^ With further increasing Mn, the average diameter increased to 8.5 ± 1.3 nm, specifically for Co_0.831_Mn_0.169_P, suggesting that growth kinetics are similar to parent CoP. Furthermore, morphology of Co_1–*x*_Mn_*x*_P NCs showed subtle differences compared to parent CoP NCs. All Mn-doped NCs exhibit pseudospherical morphology with pointy, rough-cornered surfaces, which are more visible for Co_0.909_Mn_0.091_P and Co_0.870_Mn_0.130_P NCs. Binary CoP NCs exhibit spherical particles with well-defined borders. When the Mn content is low (e.g., Co_0.947_Mn_0.053_P), NCs display narrow size dispersity. However, when the Mn content is high (e.g., Co_0.831_Mn_0.169_P), NCs exhibit slightly higher polydispersity. This suggests that the isotropic NC growth is impacted by the concentration of the Mn_2_(CO)_10_ precursor. Overall, all Co_1–*x*_Mn_*x*_P compositions exhibit similar pseudospherical morphology with pointy, rough-cornered surfaces, enabling a systematic investigation of the HER activity as a function of composition without convoluting factors from differences in morphology and crystalline phase.

**Figure 3:**
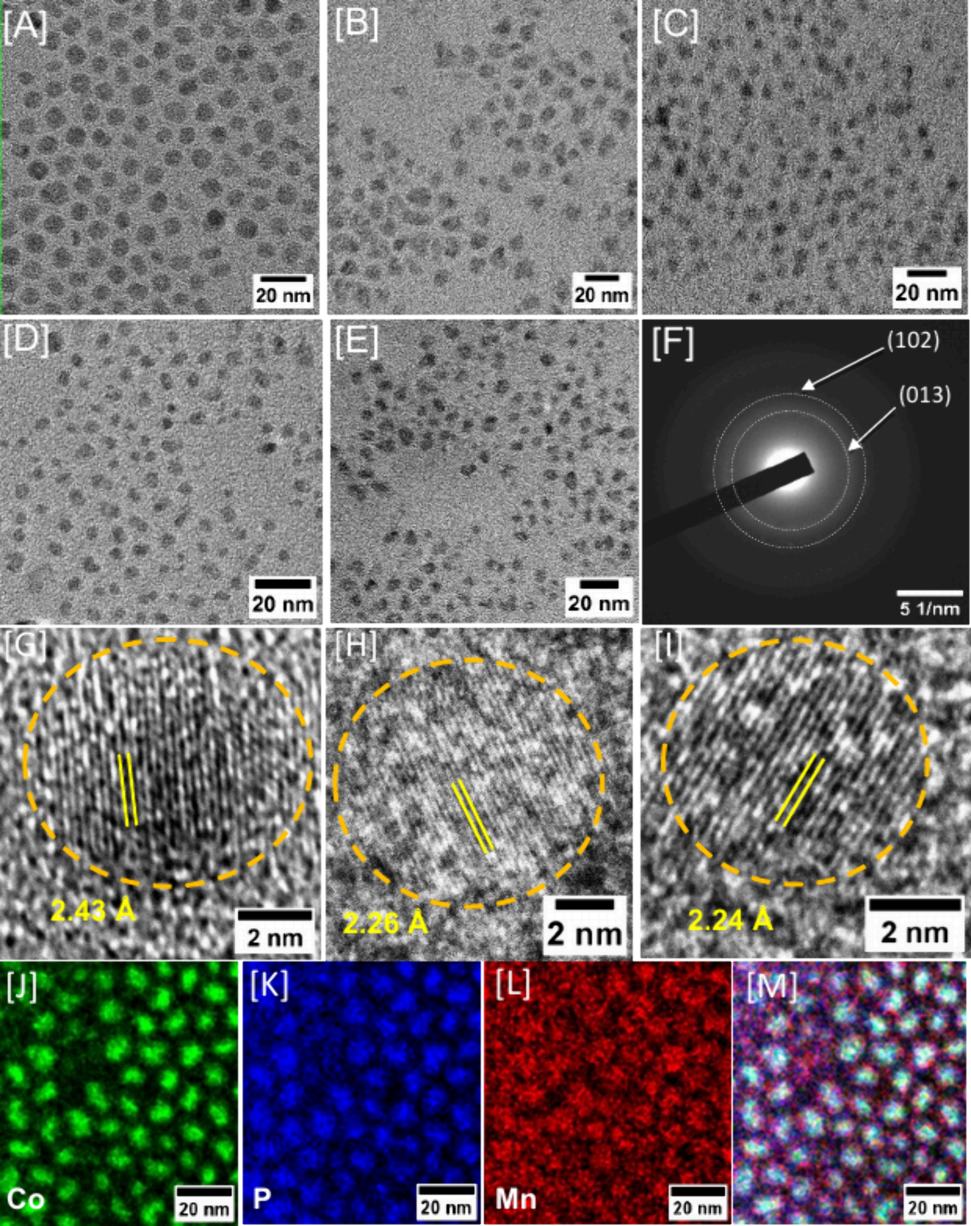
Representative TEM images of Co_1–*x*_Mn_*x*_P NCs with x = [A] 0, [B] 0.053, [C] 0.091, [D] 0.130, and [E] 0.169 compositions. [F] A representative SAED pattern of Co_0.831_Mn_0.169_P NCs. HRTEM images of Co_1–*x*_Mn_*x*_P NCs with x = [G] 0, [H] 0.091, and [I] 0.130 compositions, demonstrating lattice spacings that can be indexed to orthorhombic (102) reflection. STEM-EDS elemental maps of Co_0.831_Mn_0.169_P NCs: [J] Co, [K] P, [L] Mn, and [M] an overlay of all elements.

As-synthesized Co_1–*x*_Mn_*x*_P NCs exhibit high crystallinity, which is also reflected in SAED patterns and HRTEM images (Figure 3F–I). HRTEM images demonstrate lattice spacings of 2.43, 2.26, and 2.24 Å, corresponding to (102) planes of orthorhombic CoP, Co_0.909_Mn_0.091_P, and Co_0.870_Mn_0.130_P, respectively (Figure 3G-I). A slight decrease in *d*-spacing was noted with the increasing Mn content, which can be attributed to decreasing the metal d-electron count (Co → Mn), which stabilizes the MnP-type distortions and strengthens metal–phosphorus bonds, leading to shorter metal–phosphorus distances and therefore unit cell dimensions.^59^ SAED patterns of Co_0.831_Mn_0.169_P NCs display reflections corresponding to (013) and (102) planes of orthorhombic CoP (Figure 3F). STEM-EDS maps of the highest Mn containing sample, Co_0.831_Mn_0.169_P NCs, were recorded to examine the compositional homogeneity of bimetallic NCs. High-angle annular dark-field (HAADF) images and STEM-EDS maps of Co_0.831_Mn_0.169_P NCs show no signs of segregation and uniform distribution of elements throughout the particles, supporting the homogeneous solid solution behavior (Figure 3J-M). These results indicate that Co_1–*x*_Mn_*x*_P compositions produced in this study are structurally homogeneous alloys.

Surface and core oxidation states of CoP and Co_1–*x*_Mn_*x*_P NCs were investigated using XPS. The high-resolution XPS spectra of Co_0.909_Mn_0.091_P and Co_0.831_Mn_0.169_P NCs and parent CoP NCs are shown in Figure 4 and Supporting Information, Figure S4, respectively. The Co 2p spectrum of CoP NCs exhibits two prominent deconvoluted peaks at 778.8 and 793.8 eV, which can be assigned to Co–P bonds, consistent with the literature.^60,61^ The shoulders observed at 779.3 and 796.5 eV are likely caused by a native oxide (Co^2+^ or Co^3+^) that forms on the NC surface upon exposure to ambient atmosphere. The peaks observed at 782.1 and 802.7 eV are satellites corresponding to Co 2p_3/2_ and Co 2p_1/2_ regions, respectively.^62^ Similarly, the deconvoluted peaks observed at 130.1 eV (P 2p_3/2_) and 131.2 eV (P 2p_1/2_) in the P 2p region can be ascribed to core and surface phosphorus species coordinated to Co–P (phosphide) bonds (Supporting Information, Figure S4B).^60–62^ The broad peak observed at 135.0 eV indicates the presence of P–O bonds originating from surface PO_*x*_ species formed during handling of samples in ambient air.

**Figure 4:**
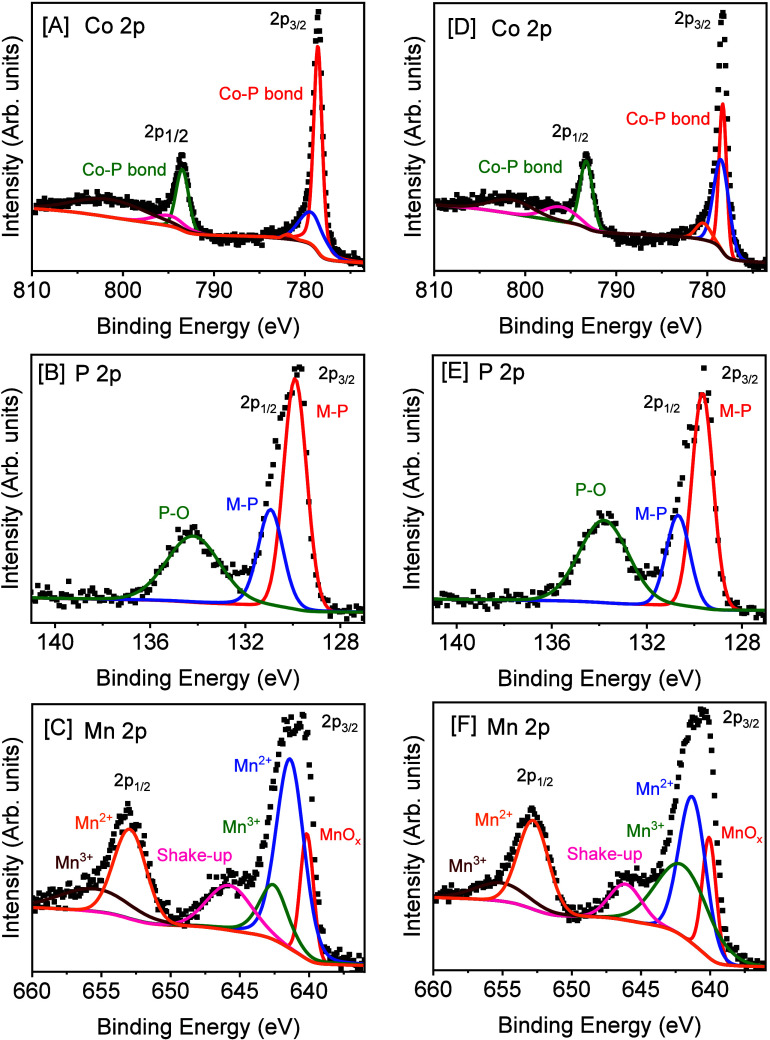
XPS spectrum of Co_0.909_Mn_0.091_P NCs displaying (A) Co 2p, (B) P 2p, and (C) Mn 2p regions along with the XPS spectrum of Co_0.831_Mn_0.169_P NCs showing (D) Co 2p, (E) P 2p, and (F) Mn 2p regions. The spectral data are represented by square symbols, and colored lines are fitted deconvolutions. Samples were annealed at 450 °C for 2 h under the 5% H_2_:Ar flow.

In contrast, the binding energies of Co and P species in Co_1–*x*_Mn_*x*_P NCs are shifted to lower energies compared to binary CoP NCs. For instance, the Co 2p region of the Co_0.909_Mn_0.091_P and Co_0.831_Mn_0.169_P NCs shows a shift of deconvoluted Co–P peaks to lower energies by ∼0.3 and 0.5 eV, respectively upon Mn incorporation (Figures 4A and 4D). These shifts indicate that the Co species in Co_1–*x*_Mn_*x*_P NCs exhibit a higher electron density than CoP NCs. Likewise, the P 2p binding energies of metal-phosphide (M-P) bonds are shifted to lower energies by ∼0.3 and 0.4 eV for Co_0.909_Mn_0.091_P and Co_0.831_Mn_0.169_P NCs (Figures 4B and 4E), respectively, suggesting that the P species in Co_1–*x*_Mn_*x*_P NCs exhibit a higher negative charge compared to CoP NCs. The shifting of Co 2p and P 2p peaks to lower binding energies can be expected because of the lower electronegativity of Mn (1.55) compared to Co (1.88). Since Mn is less electronegative, it could donate electron density to Co, making it more electron-rich. This could shift Co 2p binding energies to lower energies by increasing the electron density of Co species. Upon admixture of Mn, the binding energy of P 2p also lowered compared to parent CoP, suggesting that electrons were transferred from Co to P.^61^ This modified surface polarization could facilitate effective electron transfer during the HER. The notable deconvoluted peaks observed at 641.4 and 653.0 eV in the Mn 2p region of Co_0.909_Mn_0.091_P NCs (Figure 4C) can be attributed to Mn^2+^ species; the peaks at 642.7 and 655.8 eV are indicative of Mn^3+^ species; and the peak at 645.9 eV can be attributed to a shakeup satellite of Mn^2+^ species.^37,61,63,64^ A shakeup satellite peak of Mn^2+^ species was observed in the Mn 2p_3/2_ region because of the photoemission process that stimulates electrons, resulting in multiplets and also a satellite peak.^64^ The peak at 640.1 eV likely originates from oxidized Mn species (MnO_*x*_), consistent with the literature.^61,65^ With increasing the Mn concentration, both Mn^2+^ and Mn^3+^ peaks slightly shift to lower binding energies, and the shakeup satellite peak of Mn^2+^ shifts to higher binding energies, as observed in XPS spectrum of Co_0.831_Mn_0.169_P NCs (Figure 4F).

### Electrocatalytic Activity of CoP and Co_1–*x*_Mn_*x*_P NCs for the HER

The HER activity and stability of CoP and Co_1–*x*_Mn_*x*_P NCs and commercial Pt/C (10% wt.) catalysts were investigated in alkaline (1 M KOH) electrolytes via LSV. The long-chain organic ligands, such as OLA and TOP, decrease the electrocatalytic activity of colloidal NCs by obstructing electrical transport and reducing available surface sites for the HER. Hence, working electrodes were annealed in a tube furnace at 450 °C for 2 h in 5% H_2_/Ar atmosphere to eliminate residual surface ligands. This annealing step enhances the ohmic contact between Co_1–*x*_Mn_*x*_P NCs and Ti foil, thereby improving the catalyst adhesion to the substrate. FT-IR spectra and PXRD patterns of Co_0.909_Mn_0.091_P NCs, before and after annealing, validate the removal of surface ligands with no changes in the crystal structure (Supporting Information, Figures S2 and S5). This was confirmed by the loss of C–H stretches at 2921 and 2849 cm^–1^, the C–N stretch at 1462 cm^–1^, and the C–P stretch at 1068 cm^–1^, along with weakening of the C–H bending peak at 2160 cm^–1^ upon annealing. Moreover, the residual carbon content in the as-synthesized NCs was minimal, consistent with an ∼1.857% weight loss obtained from TGA analysis for Co_0.909_Mn_0.091_P NCs (Supporting Information, Figure S6A). Similarly, the Raman spectra of the annealed Co_0.909_Mn_0.091_P NCs display D- (1350 cm^–1^) and G-band (1580 cm^–1^) features,^66^ corresponding to amorphous carbon (Supporting Information, S6B). Since all samples were produced and annealed under identical conditions, the amorphous carbon content in the annealed NCs is expected to be similar. The Ti substrate exhibits a negligible HER activity (η_–10_ = 440.70 mV), and the corresponding LSV curve remained flattened up to −0.7 V. Meanwhile, binary CoP NCs deposited on Ti foils displayed η_–10_ of 166.70 mV, which is slightly lower than the literature reported values (173–209 mV).^42,43^ Upon Mn doping, a significant variation in HER activity was noted, consistent with ΔG_H_ calculations (Figure 1). It was found that when the Mn content is ≤ 4% (x = 0.077) or ≥ 5.1% (x = 0.091), the η_–10_ values were larger compared to CoP NCs, indicating a decrease in HER activity (Figure 5A and Table 3). Specifically, Co_0.962_Mn_0.038_P, Co_0.947_Mn_0.053_P, Co_0.870_Mn_0.130_P, and Co_0.831_Mn_0.169_P compositions displayed η_–10_ of 230.44, 210.73, 292.96, and 201.63 mV, respectively, suggesting a pronounced decrease in HER activity. However, Co_0.909_Mn_0.091_P (x = 5.092%) and Co_0.923_Mn_0.077_P (x = 4.028%) NCs showed η_–10_ of 136.29 and 161.65 mV, respectively, demonstrating a notable increase in HER activity in comparison to parent CoP NCs (Figure 5A). TEM analysis revealed that the samples with ∼4–5% Mn exhibit smaller diameters with pointy, rough-cornered surfaces. These characteristics, along with optimal Mn doping (x = ∼4–5%), likely contributed to increased HER activity of both samples. These findings are consistent with our composition-dependent ΔG_H_ calculations that predicted the lowest overall ΔG_H_ for x = 3.13% composition, followed by a prominent increase in overall ΔG_H_ with increasing Mn composition (6.26–9.39%), as illustrated in Table 1 and Supporting Information, Figure S1.

**Figure 5:**
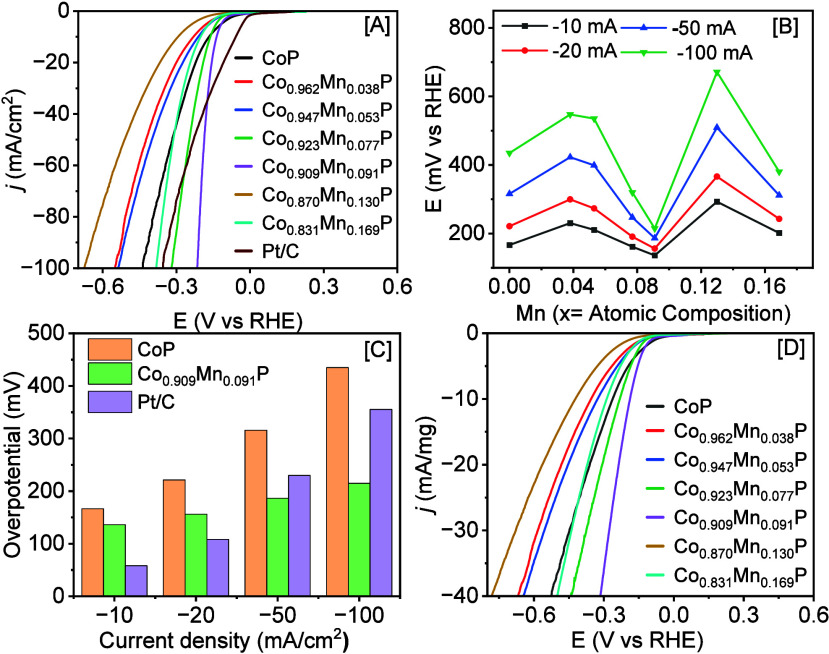
[A] *iR-*corrected HER polarization plots of CoP and Co_1–*x*_Mn_*x*_P NCs along with the commercial Pt/C (10% wt.) catalyst in 1 M KOH, normalized by the geometrical surface area. [B] Variation of overpotentials at *j* = −10, −20, −50, and −100 mA/cm^2^ as a function of Mn composition. [C] A bar graph illustrating the variation of HER overpotentials at different current densities for CoP, Co_0.909_Mn_0.091_P, and commercial Pt/C catalysts. [D] Mass-normalized LSV plots of CoP and Co_1–*x*_Mn_*x*_P NCs with variable compositions.

**Table 3:** Comparison of Overpotentials, Tafel Slopes, and ECSAs of Co_1–*x*_Mn_*x*_P Catalysts

	Overpotentials (mV) at different current densities					
Samples	η_–10_	η_–20_	η_–50_	η_–100_	Tafel slope (mV/dec)	ECSA (cm^2^)
CoP	166.70	221.30	315.78	434.91	129.02	0.260
Co_0.962_Mn_0.038_P	230.44	299.79	422.84	547.34	152.24	0.177
Co_0.947_Mn_0.053_P	210.73	273.48	398.93	535.36	147.46	0.191
Co_0.923_Mn_0.077_P	161.65	190.77	247.10	319.78	76.45	0.476
Co_0.909_Mn_0.091_P	136.29	156.25	186.53	214.91	65.77	0.549
Co_0.870_Mn_0.130_P	292.96	366.31	509.16	671.41	154.62	0.056
Co_0.831_Mn_0.169_P	201.63	242.91	311.66	380.38	105.27	0.213
Pt/C	58.19	108.44	229.92	355.31	62.31	0.618

Figure 5B illustrates the η values obtained at *j* = −10, −20, −50, and −100 mA/cm^2^ for Co_1–*x*_Mn_*x*_P NCs with variable Mn compositions. The highest HER active Co_0.909_Mn_0.091_P NCs required η of 136.29, 156.25, 186.53, and 214.91 mV to reach *j* = −10, −20, −50, and −100 mA/cm^2^, which are significantly lower compared to binary CoP and other Mn-doped CoP compositions. The HER activity of the highest-performing Co_0.909_Mn_0.091_P NCs was also compared with the benchmark Pt/C catalyst (Figure 5C). Although Co_0.909_Mn_0.091_P NCs exhibit a higher η_–10_ compared to Pt/C, it outperformed the HER activity of Pt/C when *j* ≥ −35 mA/cm^2^. Among all catalysts shown in Figure 5D, the mass-normalized current density of Co_0.909_Mn_0.091_P NCs is the highest, consistent with other studies. Furthermore, electrocatalytic performance of Co_1–*x*_Mn_*x*_P NCs was evaluated by measuring the ECSA to analyze the intrinsic activity of each composition. ECSA can elucidate the HER activity variation among different NC compositions since it is directly proportional to C_DL_. The C_DL_ values were derived from the CV curves within the nonfaradaic region (0.1 to 0.2 V vs RHE), as shown in Supporting Information, Figures S7 and S8. The calculated ECSA of Co_1–*x*_Mn_*x*_P NCs varies from 0.056 to 0.549 cm^2^ for x = 0.038–0.169 compositions (Table 3). The ECSA of Co_0.962_Mn_0.038_P, Co_0.947_Mn_0.053_P, Co_0.923_Mn_0.077_P, and Co_0.909_Mn_0.091_P NCs was estimated to be 0.177, 0.191, 0.476, and 0.549 cm^2^, demonstrating an increasing trend with increasing Mn composition. The ECSA of CoP NCs and the highest-performing Co_0.909_Mn_0.091_P NCs was 0.256 and 0.549 cm^2^ respectively, which suggests that optimal Mn doping (5.092%) can substantially enhance the number of exposed surface sites for electrocatalysis. Therefore, the improved HER activity of Co_0.909_Mn_0.091_P NCs can also be attributed to a higher ECSA achieved with optimal Mn doping.^67^ Nevertheless, the ECSA values of Co_0.870_Mn_0.130_P and Co_0.831_Mn_0.169_P NCs were decreased to 0.056 and 0.213 cm^2^, respectively, with increasing Mn content above 5.092%. To distinguish the effects of surface area from intrinsic catalytic activity, HER polarization curves were normalized with the ECSA to compute specific activity, *j*/ECSA, as shown in Supporting Information, Figure S9. The highest performing Co_0.909_Mn_0.091_P NCs still exhibit the highest HER activity suggesting that the performance improvement is due not only to increased surface area but also the improved intrinsic activity caused by Mn incorporation. The highest active Co_0.909_Mn_0.091_P catalyst outperformed the Pt/C standard above 204.60 mV after specific activity normalization. Overall, the Co_0.909_Mn_0.091_P composition displayed the highest HER activity and the greatest potential as an earth-abundant catalyst for alkaline HER specifically at industrially relevant, higher *j* values (*j* ≥ −35 mA/cm^2^).

Tafel slopes were estimated from the polarization curves to investigate the reaction kinetics (Figure 6A-B). Typically, lower Tafel slopes indicate improved reaction kinetics and consequently higher HER activity. The computed slopes of Co_1–*x*_Mn_*x*_P NCs vary between 65.77 and 154.62 mV/dec, indicating a Volmer-Heyrovsky mixed HER mechanism, where the Volmer step contributes significantly to the HER, and it is the primarily rate-determining step of all catalysts. The binary CoP NCs displayed slower HER kinetics, as indicated by a Tafel slope of 129.02 mV/dec, in comparison to the highest-performing Co_0.909_Mn_0.091_P catalyst (65.77 mV/dec). In contrast, commercial Pt/C displayed a Tafel slope of 62.31 mV/dec. The slope values of optimally doped Co_0.909_Mn_0.091_P NCs and commercial Pt/C are closer to each other, suggesting that both catalysts exhibit comparable HER kinetics and mechanisms. It is likely that the adsorption of the hydrogen species on the Co_1–*x*_Mn_*x*_P NCs is negatively impacted when the Mn content is either below or above x = 0.091. The optimal dopant composition of Co_0.909_Mn_0.091_P NCs likely accelerates the rate-determining Volmer step (H_2_O + e^–^ → H_ads_ + OH^–^); however, the subsequent Heyrovsky step (H_ads_ + H_2_O + e^–^ → H_2_ + OH^–^) to generate H_2_ still proceeds at a slower rate. The binding energies of the metal-phosphide (M-P) in Co 2p and P 2p regions are lowered upon Mn doping, suggesting a more negatively charged P site and an enhanced electron density on Co sites (Figure 4). The optimal negatively charged P is expected to facilitate proton capture (H_ads_), in accordance with the improved Volmer-dominated kinetics observed for Co_0.909_Mn_0.091_P NCs. Additionally, the presence of mixed Mn oxidation states (Mn^2+^/Mn^3+^) suggests a redox surface environment, leading potentially to enhanced HER kinetics. In Figure 6C and Supporting Information, Figure S10 and Table S2, we examined the charge transfer resistance (R_ct_) and solution resistance (R_s_) of phosphide catalysts. The computed R_ct_ values of Co_0.962_Mn_0.038_P, Co_0.947_Mn_0.053_P, Co_0.923_Mn_0.077_P, Co_0.909_Mn_0.091_P, Co_0.870_Mn_0.130_P, and Co_0.831_Mn_0.169_P NCs were 56.2, 68.7, 130.4, 31.2, 16.7, 150.7, and 58.9 Ω, respectively, from the fitted Nyquist plots with an equivalent circuit illustrated in the inset of Figure 6C. Among all phosphides, the highest HER active Co_0.909_Mn_0.091_P NCs demonstrated the lowest R_ct,_ suggesting enhanced charge transfer between the NCs and reactive species, leading to improved HER performance (Figure 6D).

**Figure 6:**
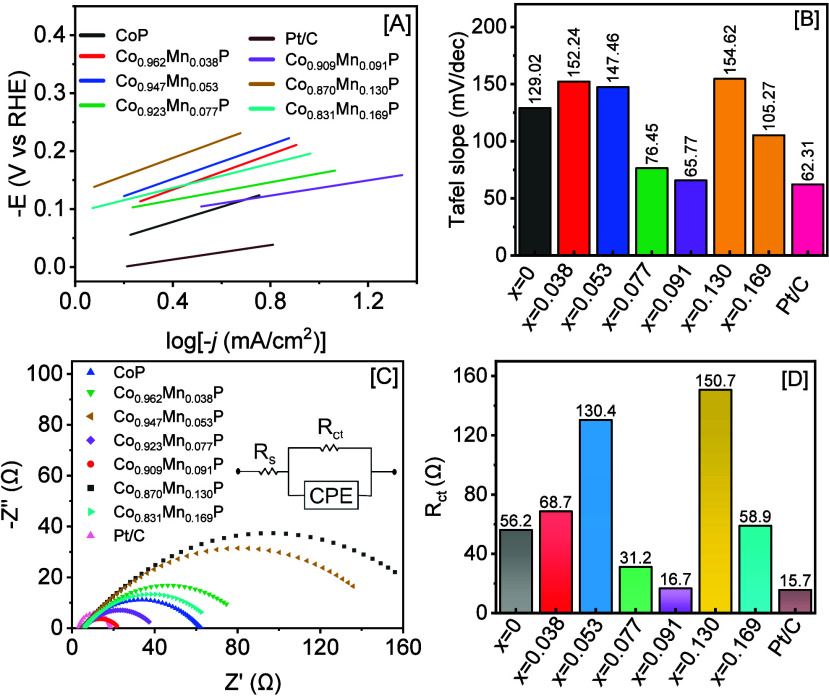
[A] Tafel plots of Co_1–*x*_Mn_*x*_P NCs (x = 0–0.169) and the commercial Pt/C (10% wt.) catalyst derived using HER polarization curves, along with [B] corresponding slope values. [C] Electrochemical impedance spectroscopy plot of CoP and Co_1–*x*_Mn_*x*_P NCs with an equivalent circuit shown in the inset. [D] A plot illustrating the variation of charge transfer resistance, R_ct_, as a function of the Mn composition.

The stability and durability of Co_1–*x*_Mn_*x*_P NCs and commercial Pt/C catalysts for HER were examined using the 10 h long chronopotentiometry (CP) study at *j* = −10 mA/cm^2^ in 1 M KOH (Figure 7A). The CP curve of commercial Pt/C shows a gradual increase in η_–10_ over time, suggesting a decrease in HER performance. For CoP NCs, η_–10_ decreases slightly within the first hour, suggesting an increase in HER performance. After that, a rapid increase in η_–10_ was observed suggesting an overall decrease in HER performance. Conversely, the highest active Co_0.909_Mn_0.091_P NCs demonstrated exceptional stability and a modest decrease in η_–10_ throughout the CP test. To better grasp the influence of materials’ stability on HER activity and kinetics, Figures 7B and 7C compare the polarization curves and Tafel slopes before and after CP. After CP, the HER activity of binary CoP NCs was decreased by 16.13, 6.62, and 4.71% at η_–10_, η_–50_, and η_–100_, respectively (Supporting Information, Table S3). This was accompanied by an increase of η_–10_ from 166.70 to 193.59 mV, an increase of η_–50_ from 315.78 to 336.68 mV, and an increase of η_–100_ from 434.92 to 455.40 mV (Figures 7B and 7D), meaning that CoP NCs are moderately stable at lower *j* values (−10 mA/cm^2^) yet exhibit somewhat enhanced stability at higher *j* values (−50 and −100 mA/cm^2^). In contrast, commercial Pt/C showed poor stability at lower *j* values but better stability at higher *j* values, as evidenced by an increase of η_–10_ from 58.20 to 89.85 mV (54.38% increase), an increase of η_–50_ from 229.92 to 242.76 mV (5.58% increase), and an increase of η_–100_ from 355.31 to 386.97 mV (8.91% increase). In 1 M KOH, the highest-performing Co_0.909_Mn_0.091_P NCs showed remarkable stability, with a barely noticeable shift in η values during 10 h of continuous HER both at low and high current densities. The η_–10_ increased from 136.29 to 144.23 mV, the η_–50_ increased from 186.53 to 194.45 mV, and the η_–100_ increased from 214.91 to 221.97 mV, accounting for 5.83%, 4.25%, and 3.29% increases, respectively. Postcatalytic Tafel slopes were derived from polarization curves to investigate changes in reaction kinetics. For Pt/C, the Tafel slope increased from 62.31 to 85.86 mV/dec, corresponding to a 37.79% increase. This suggests that Pt/C demonstrates slower HER kinetics and lower catalytic efficiency after the CP test. Conversely, the Tafel slope of CoP NCs showed a moderate increase from 129.02 to 140.41 mV/dec (8.83% increase) indicating a somewhat higher stability compared to commercial Pt/C. In contrast, the highest-active Co_0.909_Mn_0.091_P NCs showed the lowest slope increase, from 65.77 to 68.91 mV/dec, corresponding to an ∼4.77% increase after CP. To investigate the long-term stability, a 24 h CP test was also conducted with the highest performing Co_0.909_Mn_0.091_P catalyst at *j* = −10 mA cm^–2^ (Supporting Information, Figure S11A). The post-CP polarization curve indicates a modest degradation, with η_–10_ increasing from 136.29 to 152.94 mV (12.21%), η_–50_ increasing from 186.53 to 204.83 mV (9.82%), and η_–100_ increasing from 214.91 to 237.88 mV (10.69%), respectively (Supporting Information, Figure S11B). These data suggest that with appropriate heteroatom doping, both the HER activity and stability of CoP NCs can be significantly enhanced.

**Figure 7:**
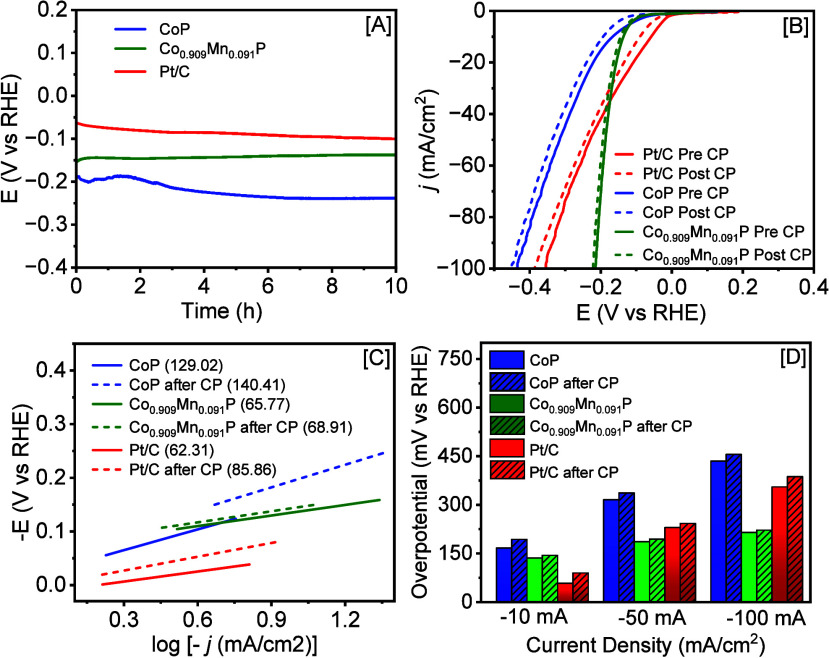
[A] Chronopotentiometry plots of binary CoP and Co_0.909_Mn_0.091_P NCs along with the commercial Pt/C catalyst recorded at *j* = −10 mA/cm^2^ in 1 M KOH. [B] and [C] illustrate the polarization curves and the corresponding Tafel slopes before (solid line) and after (dotted line) the chronopotentiometry study, respectively. [D] A bar diagram illustrating the changes in η at *j* = −10, −50, and −100 mA/cm^2^ before and after the chronopotentiometry test.

After 24 h of CP study, Co_0.909_Mn_0.091_P NCs were recovered to investigate potential changes in physical characteristics. PXRD patterns of Co_0.909_Mn_0.091_P NCs exhibit reflections corresponding to orthorhombic CoP with minor surface oxidation (Supporting Information, Figure S12A-B), suggesting that the crystal structure is still maintained after prolonged HER. TEM images of Co_0.909_Mn_0.091_P NCs show no change in morphology after CP (Supporting Information, Figure S12C–D). Consistent with PXRD, XPS spectra recorded from postcatalytic Co_0.909_Mn_0.091_P NCs indicate peaks corresponding to the Co–P bond (779.0 and 793.5 eV) and oxidized Co^2+^/Co^3+^ species at 780.7 and 796.5 eV because of the exposure to air and alkaline electrolytes (Supporting Information, Figure S13). In the P 2p region, strong peaks corresponding to the metal-phosphide (M-P) bond were observed at 129.8 and 130.8 eV in addition to the oxidized phosphorus (P–O) peak at 133.4 eV, in agreement with the minor Co_3_O_4_ impurities detected in PXRD patterns. Overall, postcatalytic characterizations support the preservation of the orthorhombic CoP structure and original morphology of Co_0.909_Mn_0.091_P NCs with minor oxidation after 24 h of continuous HER (Supporting Information, Figure S14 and Table S4).

## Conclusions

This work reports a comprehensive computational and experimental investigation of Mn-doped CoP NCs as efficient, earth-abundant catalysts for the HER in alkaline electrolytes. DFT calculations identified Mn as an effective dopant, providing optimal hydrogen adsorption energetics on the CoP (011) surface. A systematic computational analysis demonstrated that low-level (3.13%) Mn incorporation fine-tunes the hydrogen binding energies, balancing adsorption and desorption to achieve near-thermoneutral ΔG_H_. Guided by these predictions, a robust colloidal synthesis was developed to produce a series of Co_1–*x*_Mn_*x*_P NCs, allowing for systematic investigation of HER activity as a function of composition. As-synthesized Co_1–*x*_Mn_*x*_P NCs showed an orthorhombic structure, pointy rough-cornered morphology, and a decrease in diameter with increasing Mn composition. STEM-EDS elemental maps indicate the homogeneous solid solution behavior of Co_1–*x*_Mn_*x*_P NCs. XPS analysis of ternary NCs suggests a decrease in Co 2p and P 2p binding energies upon Mn incorporation, consistent with an increase in electron density on both sites. Electrocatalytic studies revealed that Co_0.909_Mn_0.091_P composition exhibits the highest HER activity, delivering a η_–10_ of 136.29 mV and outperforming the HER activity of the benchmark Pt/C catalyst at *j* ≥ −35 mA/cm^2^, in agreement with composition-dependent DFT studies. ECSA calculations showed a pronounced increase in active surface area and a significantly lower R_ct_ value (16.7 Ω) for Co_0.909_Mn_0.091_P NCs compared to parent CoP and other Mn-doped CoP compositions. The highest-performing Co_0.909_Mn_0.091_P NCs demonstrate exceptional stability in alkaline electrolytes, with minor increases in η_–10_ (5.83%) and the Tafel slope (4.77%) after 10 h of the HER, compared to CoP and Pt/C catalysts. Overall, through computational dopant screening and appropriate heteroatom-doping, both the HER activity and stability of earth-abundant nanostructures can be greatly enhanced, enabling the development of high-efficiency, robust catalysts.

## Supplementary Material



## Notes

Computational figures of ΔG_H1_ and ΔG_H2_ on Mn-doped CoP (011) surfaces at varying Mn concentrations; ΔG_H_ values for the first and second hydrogen adsorption on pristine Co_2_P and Cr, Fe, Mn, Mo, and V-doped Co_2_P (201) surfaces; PXRD patterns of CoP and Co_1–*x*_Mn_*x*_P NCs after annealing at 450 °C for 2 h under 5% H_2_/Ar; size histograms of Co_1–*x*_Mn_*x*_P NCs with variable Mn content; XPS spectra of binary CoP NCs illustrating the Co 2p and P 2p regions; FTIR spectra of Co_0.909_Mn_0.091_P NCs; cyclic voltammetry curves for CoP and Co_1–*x*_Mn_*x*_P NCs with variable Mn content; and scan rate vs Δ*j*_0.15 V_/2 plots for CoP and Co_1–*x*_Mn_*x*_P NCs; TGA curve and Raman spectrum of as-synthesized and annealed Co_0.909_Mn_0.091_P NCs; HER polarization curves normalized with respect to ECSA (*j*/ECSA) for CoP, Co_1–*x*_Mn_*x*_P, and Pt/C catalysts; CP plot of Co_0.909_Mn_0.091_P NCs recorded at *j* = −10 mA/cm^2^ for 24 h along with the polarization curves recorded before and after CP; PXRD patterns and TEM images of Co_0.909_Mn_0.091_P NCs before and after CP; and Co 2p, P 2p, and Mn 2p XPS spectra of Co_0.909_Mn_0.091_P NCs after 24 h CP, as-acquired and area-normalized EIS plots CoP, Co_1–*x*_Mn_*x*_P, and Pt/C catalysts; and LSV curves before and after *iR-*correction (PDF)
