# Supplementary material for: Crystal Structure and Morphology-Controlled Synthesis of Co1–x Mn x P Nanocrystals and Their Composition-Dependent Electrocatalytic Activity for the Hydrogen Evolution Reaction
Source: ACS Appl Mater Interfaces. 2026 May 16;18(20):28612–25. doi: 10.1021/acsami.5c26069 (PMC13220231; doi:10.1021/acsami.5c26069)
Supplement: Supplementary file 1 [file am5c26069_si_001.pdf]

Crystal Structure and Morphology-Controlled Synthesis of $Co_{1-x}Mn_xP$ Nanocrystals and Their Composition-Dependent Electrocatalytic Activity for Hydrogen Evolution Reaction

*Md Kawsar Alam, Danyang Wang, Jordon Baker, Ka Un Lao, Indika U. Arachchige**

Department of Chemistry, Virginia Commonwealth University, Richmond, Virginia 23284-2006, United States.

Supporting Information

*Email: iuarachchige@vcu.edu

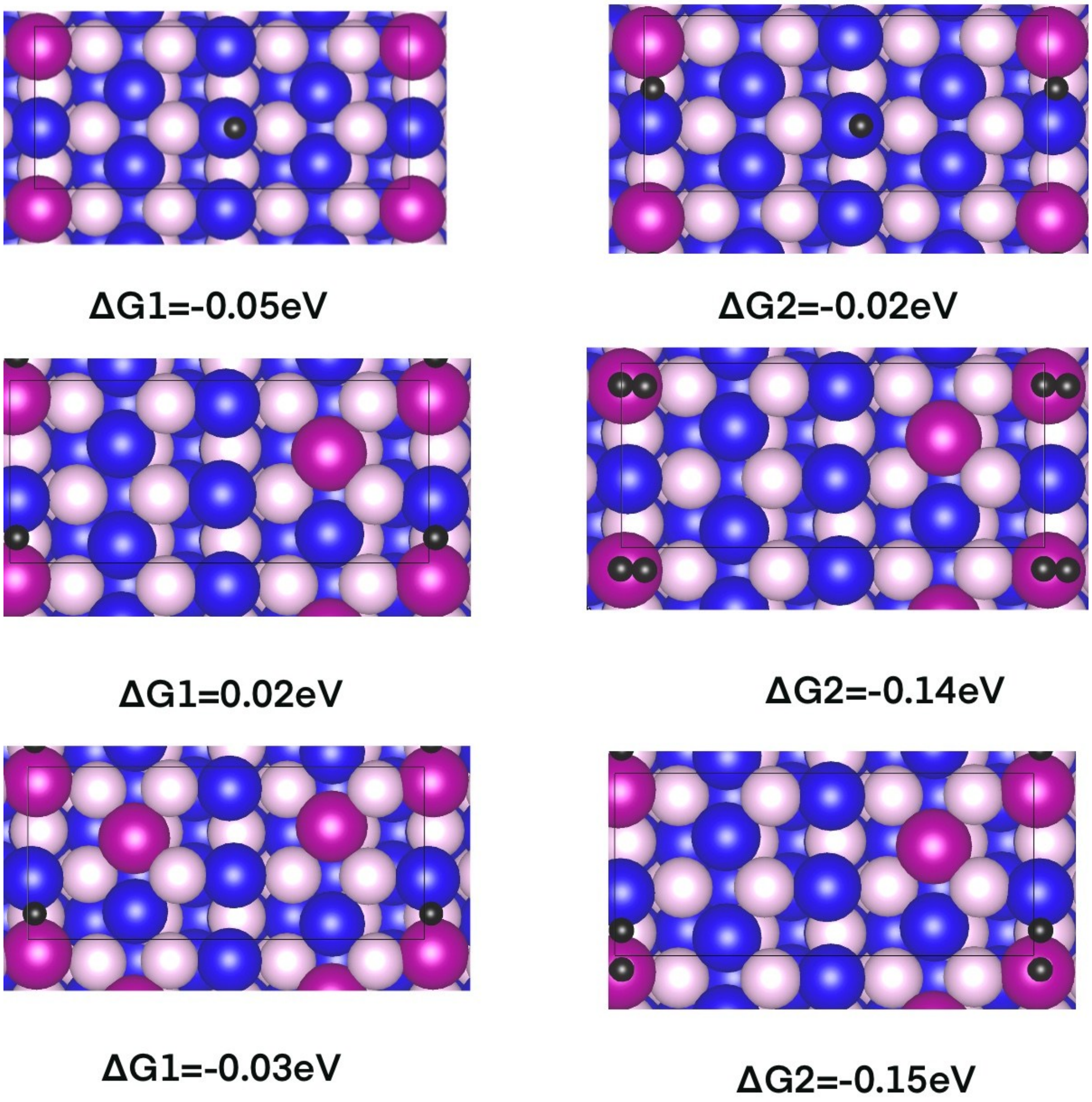

Figure S1. First and second hydrogen adsorption free energies (ΔG_{H1} and ΔG_{H2}) on Mn-doped CoP (011) surfaces at varying Mn concentrations: top (3.13%), middle (6.25%), and bottom (9.38%). Color scheme: dark blue = Co, pink = P, black = H.

Table S1. ΔG_H values for the first and second hydrogen adsorption on pristine Co_2P (201) and transition-metal-doped (Cr, Fe, Mn, Mo, and V) Co_2P (201) surfaces with Co_7P_4 termination at a dopant concentration of 3.13%.

Co_2P	ΔG_{H1} (eV)	ΔG_{H2} (eV)	$\lvert\Delta G_{H1}\rvert + \lvert\Delta G_{H2}\rvert$ (eV)
Pristine	-0.66	-0.52	1.18
Cr	-0.50	-0.35	0.85
Fe	-0.65	-0.32	0.97
Mn	-0.46	-0.43	0.89
Mo	-0.44	-0.23	0.67
V	-0.47	-0.25	0.72

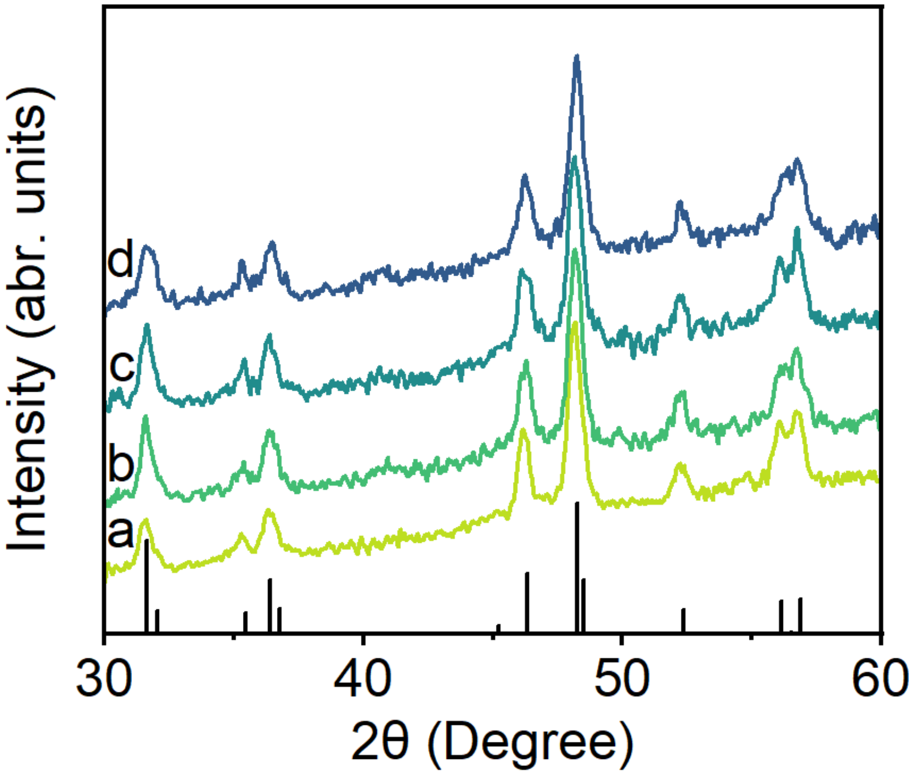

Figure S2: PXRD patterns of $Co_{1-x}Mn_xP$ NCs after annealing at 450 °C for 2 h under 5% H_2/Ar: (a) x = 0, (b) x = 0.038, (c) x = 0.053, (d) x = 0.091. The reference pattern of orthorhombic CoP (JCPDS No. 04-003-2072) is shown as vertical black lines.

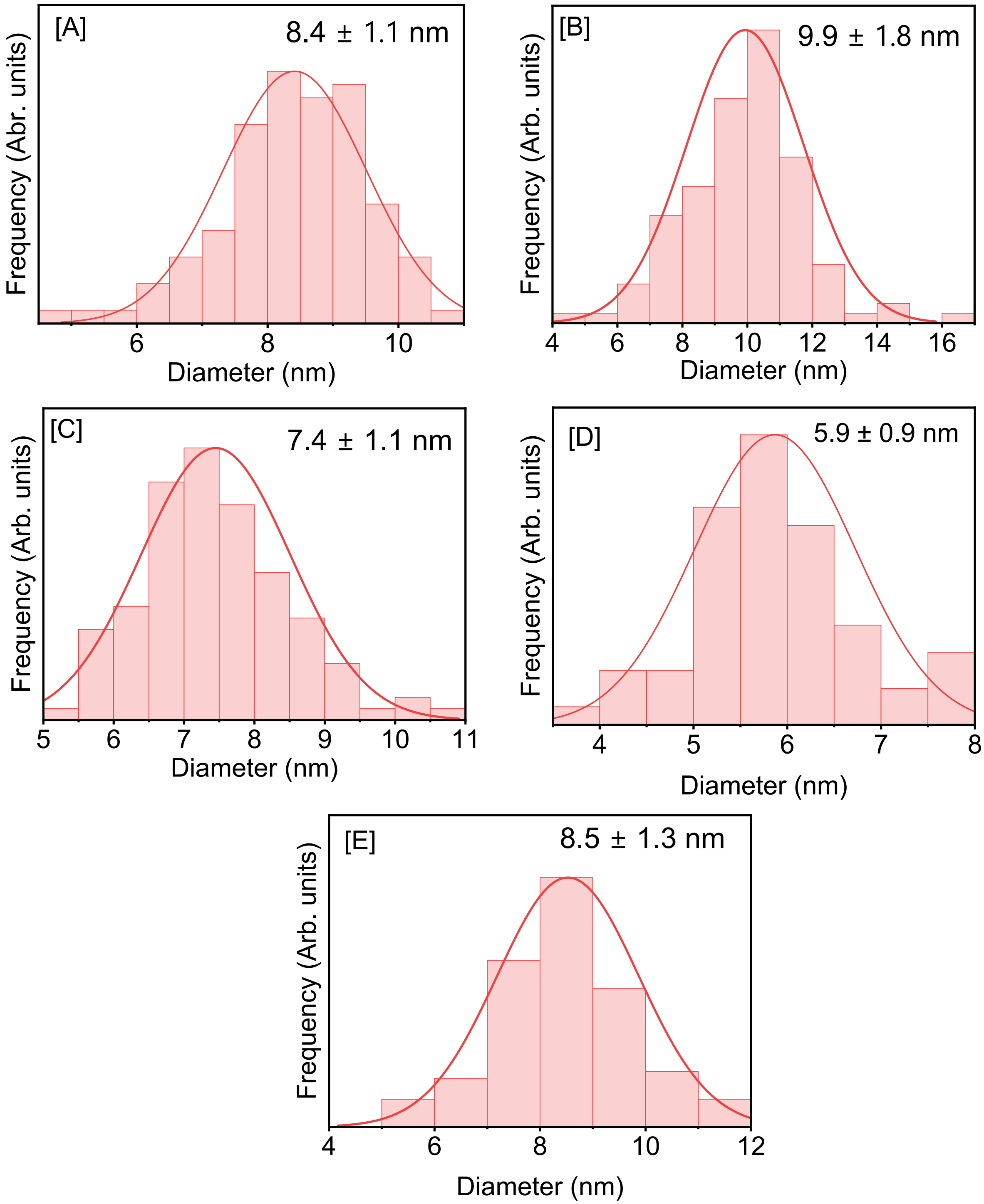

Figure S3: Size histograms of $Co_{1-x}Mn_xP$ NCs with variable Mn compositions: [A] x = 0, [B] x = 0.053, [C] x = 0.091, [D] x = 0.130, [E] x = 0.169. The average sizes computed from 150-180 NCs across LR and HRTEM images are shown within each plot.

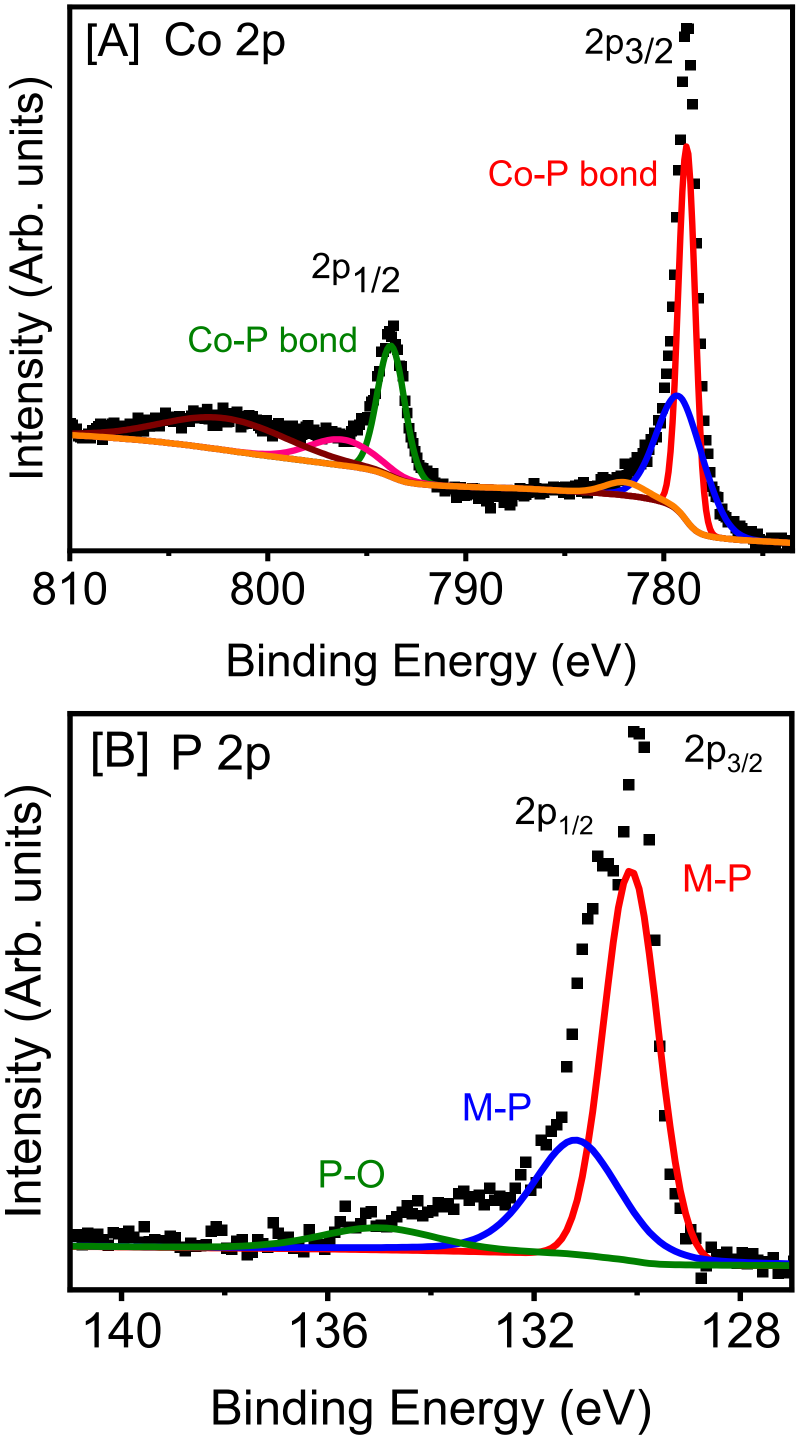

Figure S4. XPS spectra of binary CoP NCs displaying the [A] Co 2p and [B] P 2p regions. The spectral data are represented by square symbols and the colored lines are fitted deconvolutions. CoP NCs were annealed at 450°C for 2 h under 5% H_2:Ar prior to XPS analysis.

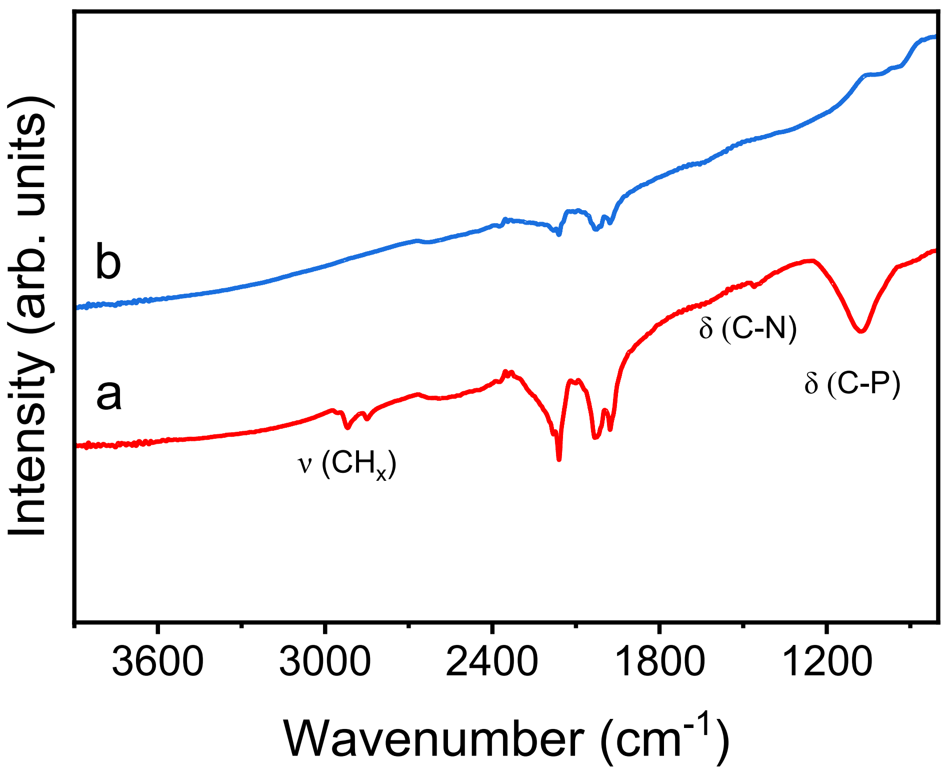

Figure S5. FTIR spectra of $Co_{0.909}Mn_{0.091}P$ NCs (a) before and (b) after annealing at 450 °C for 2 h in 5% H_2:Ar. The C-H stretches at 2921 and 2849 cm^{-1}, C-N stretch at 1462 cm^{-1}, and C-P stretch at 1068 cm^{-1} were absent after annealing, indicating that surface ligands were successfully removed. Moreover, C-H bending modes at 2160 cm^{-1} were notably weakened after annealing.

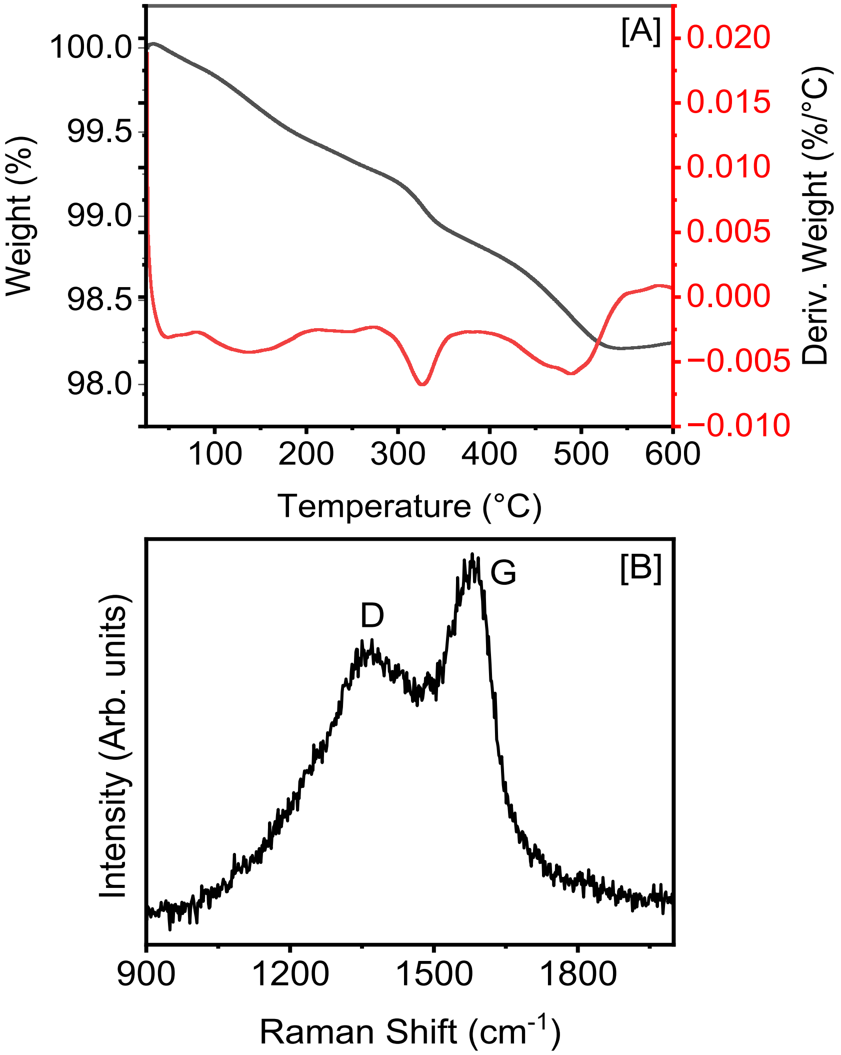

Figure S6. [A] TGA curve of as-synthesized $Co_{0.909}Mn_{0.091}P$ NCs showing minimal weight loss (1.857%). [B] Raman spectrum of $Co_{0.909}Mn_{0.091}P$ NCs annealed at 450 °C for 2 h under 5% H_2:Ar. TGA measurement was carried out under N_2 atmosphere. $Co_{0.909}Mn_{0.091}P$ NCs exhibit multistep mass losses at 136, 250, 328, and 490 °C. Physically adsorbed trace moisture and trapped washing solvents are likely responsible for the loss at ~136 °C. Progressive removal of residual surface ligands (ODE, OLA, and TOP) likely appears at ~250 and ~328 °C, with the ~328 °C step possibly representing the major ligand-decomposition process.[1] The complete burning off and desorption of the passivating surface ligands is likely responsible for the major mass loss at ~490 °C.

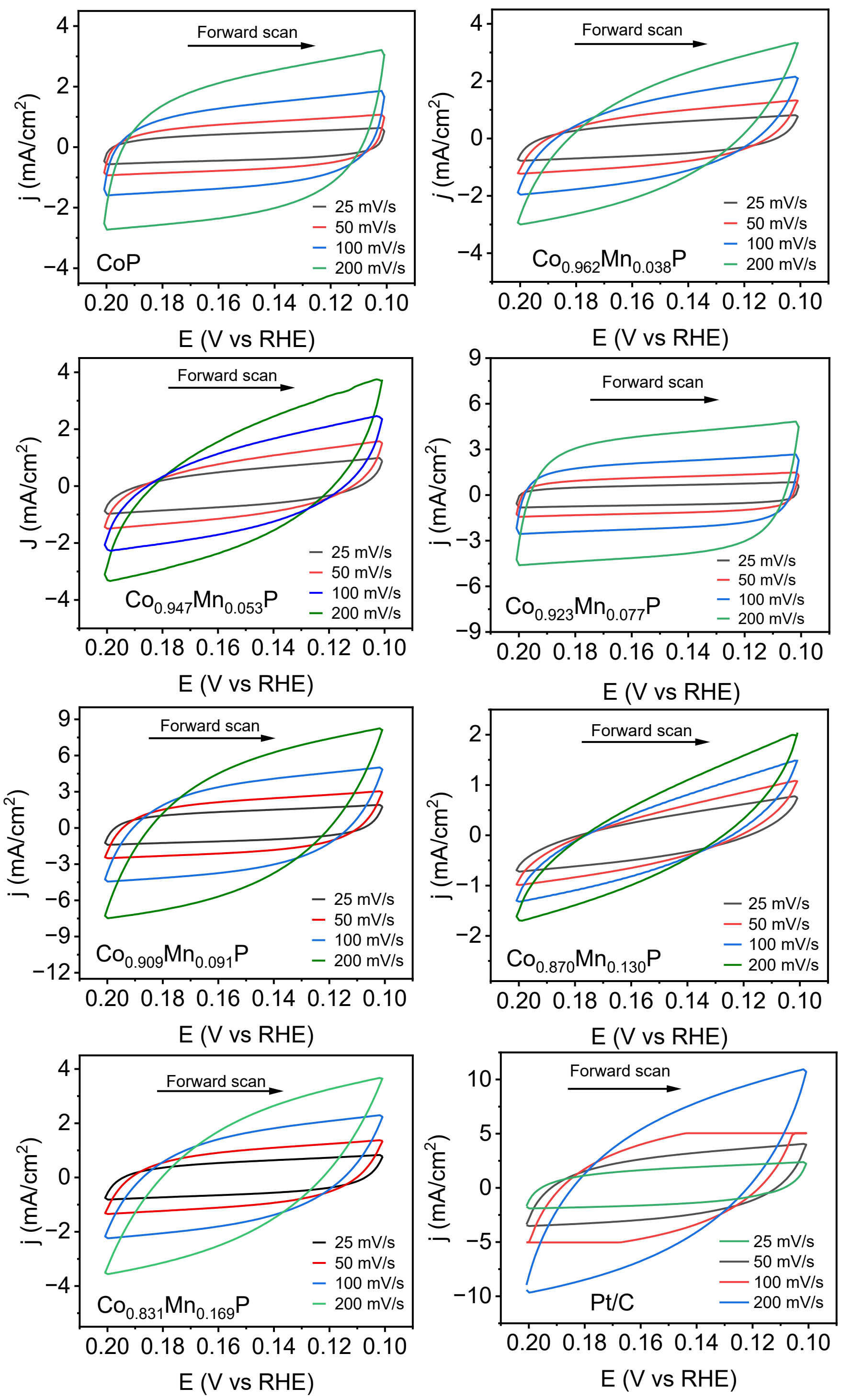

Figure S7. Cyclic voltammetry (CV) curves of CoP, $Co_{1-x}Mn_xP$ NCs (x=0.038-0.169), and commercial Pt/C catalysts in 1 M KOH. CVs were recorded by sweeping the potential from 0.1 to 0.2 V at scan rates of 25, 50, 100, and 200 mV/s.

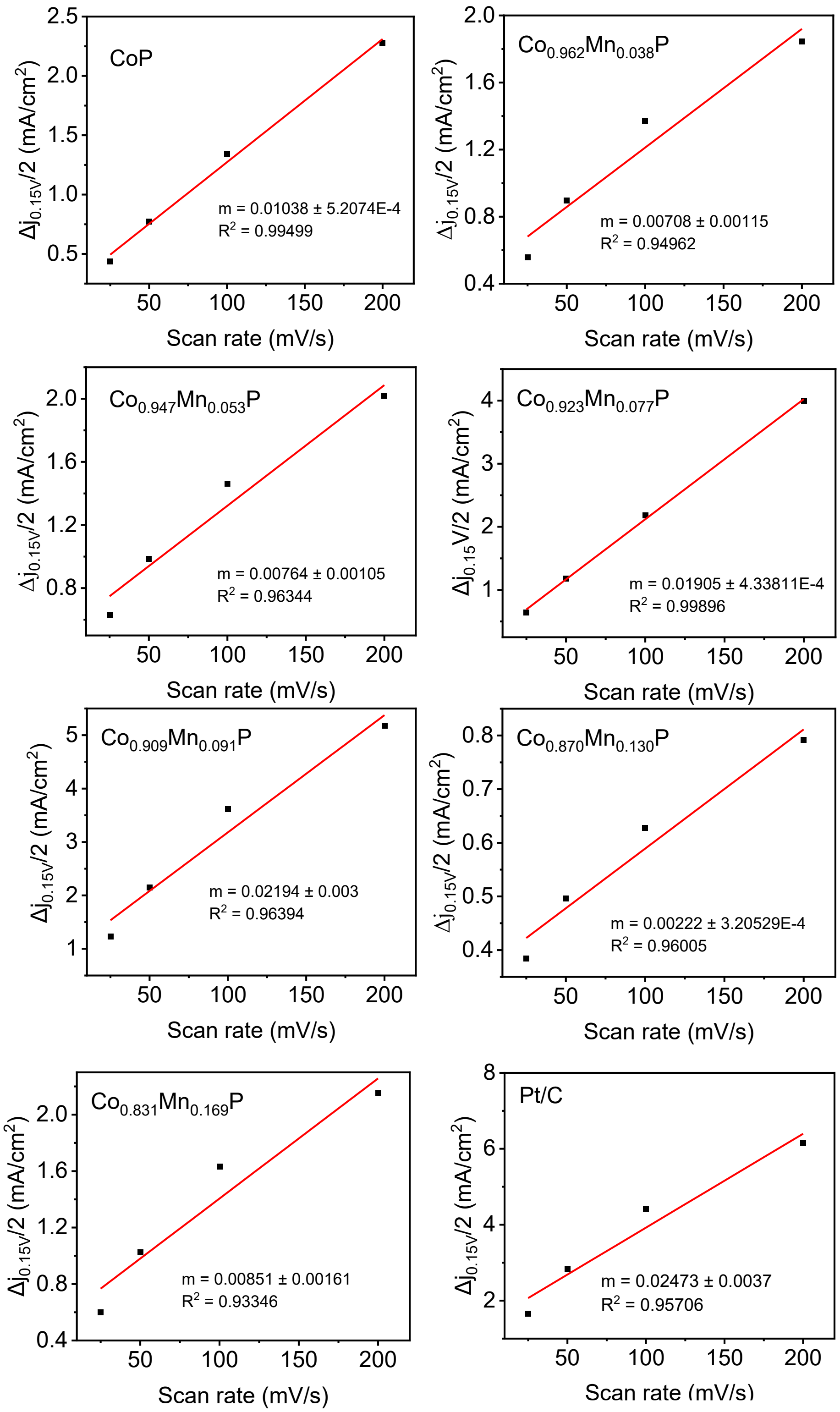

Figure S8. Scan rate vs $\Delta j_{0.15V}/2$ plots for CoP, $Co_{1-x}Mn_xP$ NCs (x=0.038-0.169), and Pt/C catalysts. These plots were linearly fitted to calculate slopes and double layer capacitance (C_{DL}).

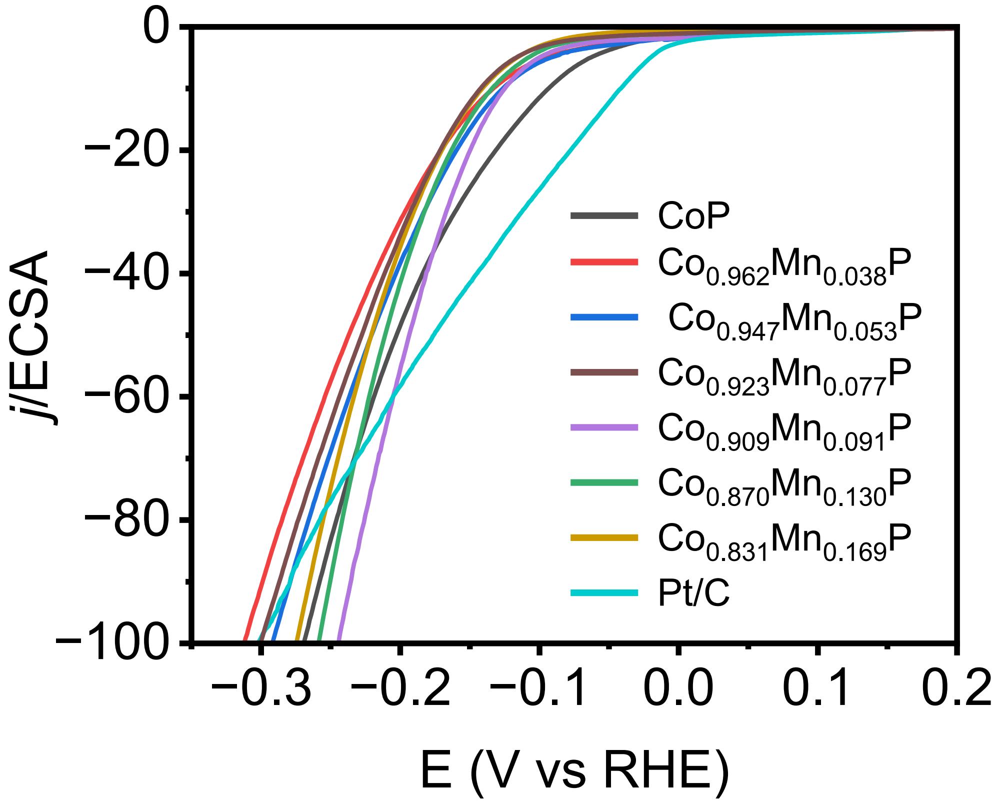

Figure S9. HER polarization curves of CoP, $Co_{1-x}Mn_xP$ NCs, and Pt/C catalysts in 1 M KOH normalized with electrochemically active surface area of each catalyst.

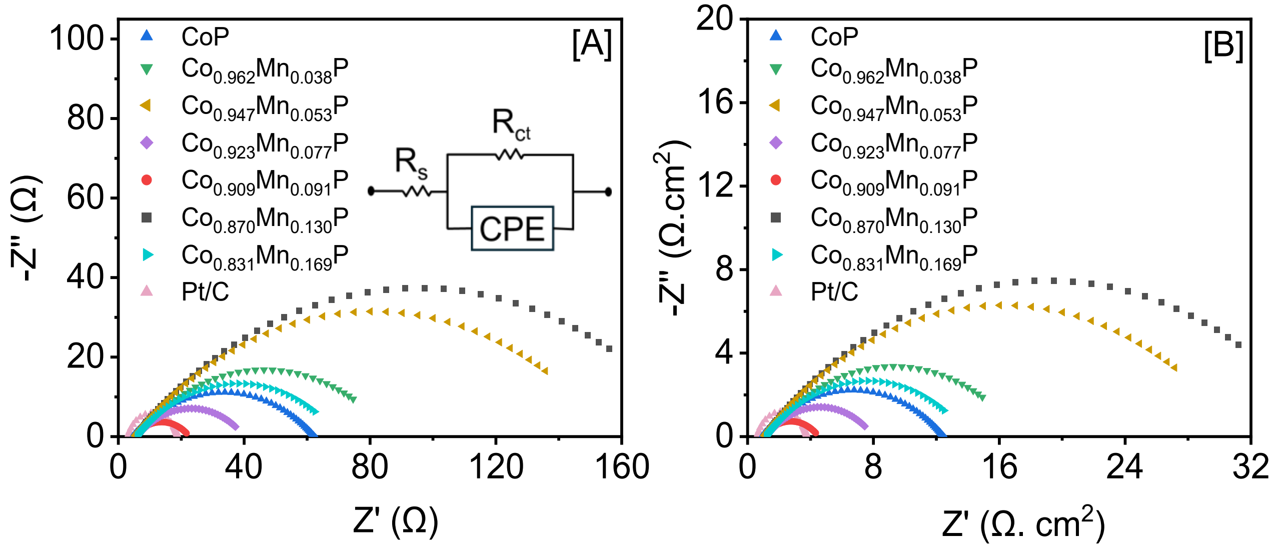

Figure S10. EIS plots of CoP, $Co_{1-x}Mn_xP$, and Pt/C catalysts in N_2-saturated 1 M KOH. [A] As-acquired impedance values and [B] impedance values normalized with respect to geometric surface area.

Table S2. Solution resistance and area-normalized solution resistance of CoP, $Co_{1-x}Mn_xP$, and Pt/C catalysts obtained from EIS in 1 M KOH.

Catalyst	R_s (Ω)	$R_{s,\ normalized}$ ($\Omega.cm^2$)
CoP	4.4	0.9
$Co_{0.962}Mn_{0.038}P$	5.9	1.2
$Co_{0.947}Mn_{0.053}P$	5.4	1.1
$Co_{0.923}Mn_{0.077}P$	6.0	1.2
$Co_{0.909}Mn_{0.091}P$	5.0	1.0
$Co_{0.870}Mn_{0.130}P$	5.3	1.1
$Co_{0.831}Mn_{0.169}P$	5.9	1.2
Pt/C	3.0	0.6

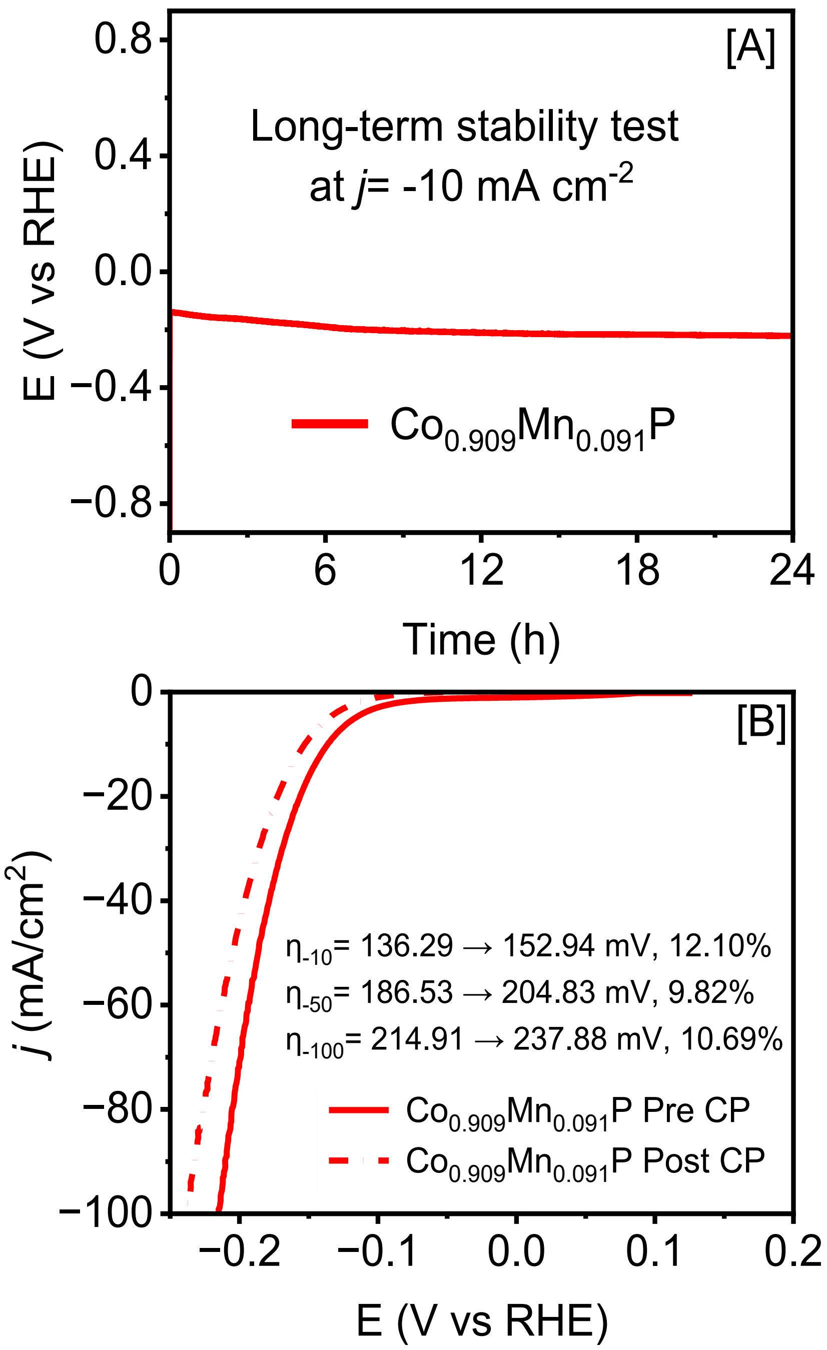

Figure S11. [A] Chronopotentiometry plot of $Co_{0.909}Mn_{0.091}P$ NCs recorded at j = -10 mA/cm^2 in N_2-saturated 1.0 M KOH along with [B] *iR*-corrected polarization curves recorded before (solid line) and after (dotted line) chronopotentiometry test, respectively.

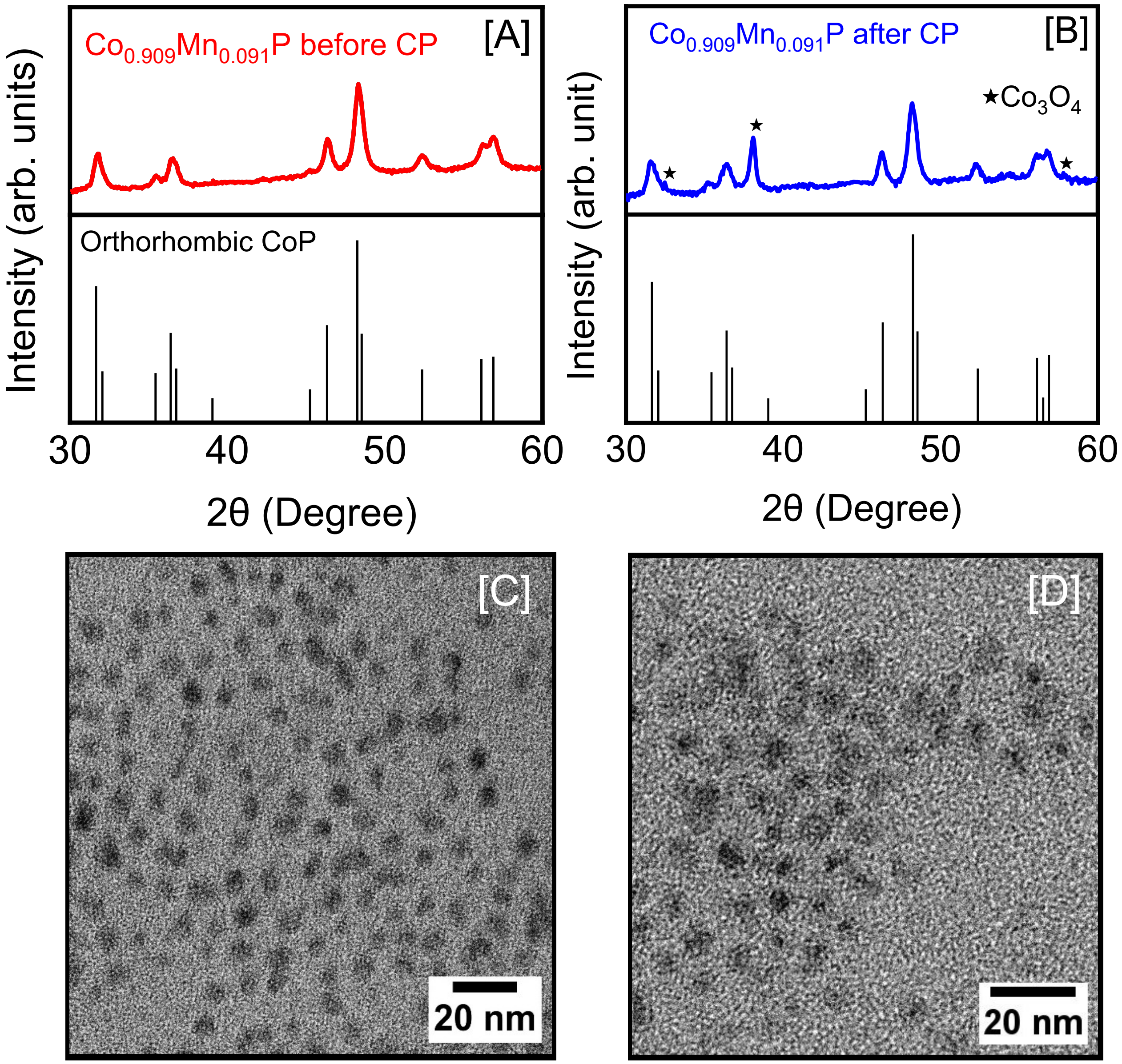

Figure S12. PXRD patterns of $Co_{0.909}Mn_{0.091}P$ NCs [A] before and [B] after a 24 h CP. The ICCD PDF overlays of orthorhombic CoP (JCPDS No. 04-003-2072) are shown as vertical black lines. [C] and [D] represent TEM images of $Co_{0.909}Mn_{0.091}P$ NCs before and after 24 h CP, respectively.

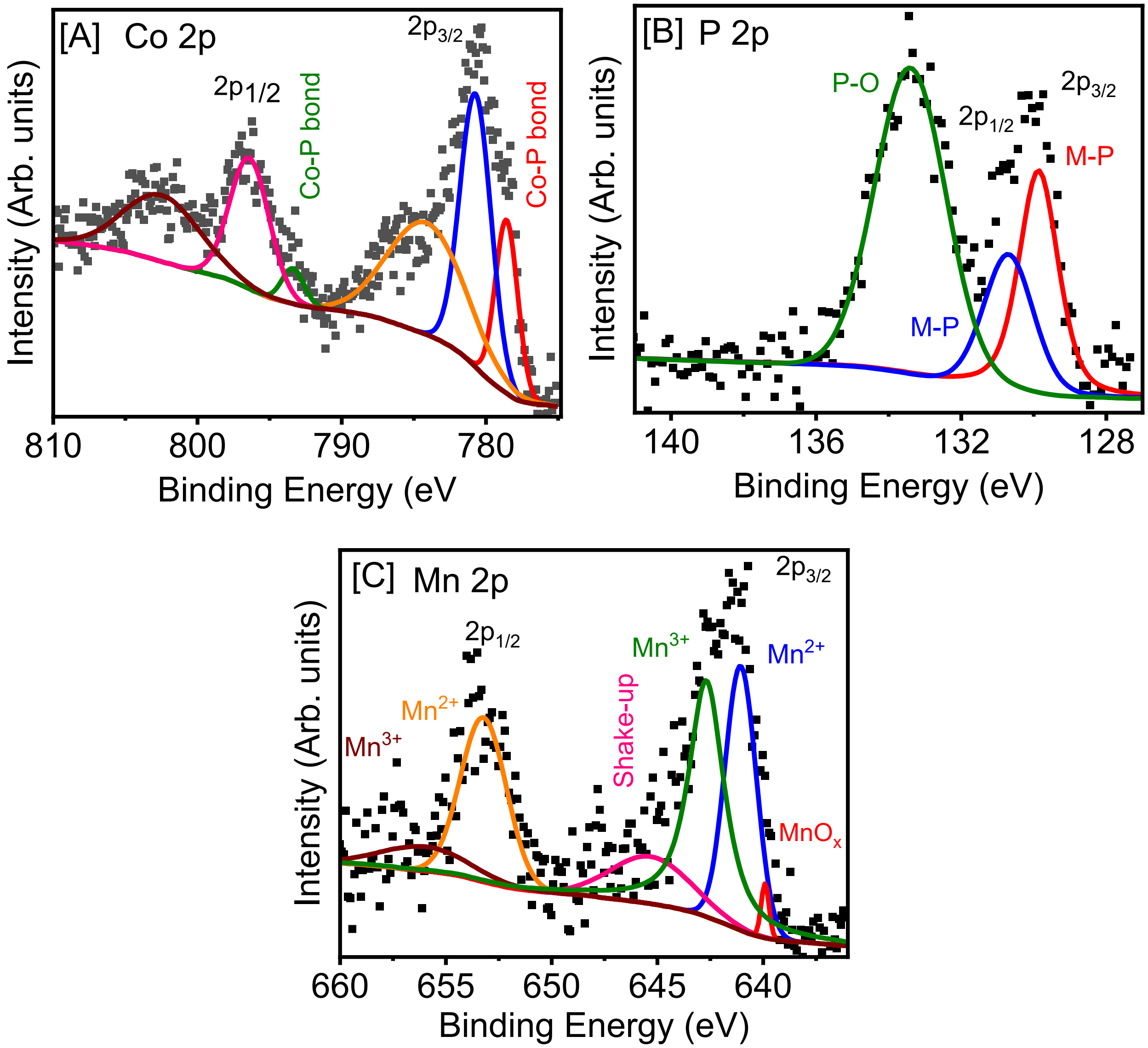

Figure S13. XPS spectrum of $Co_{0.909}Mn_{0.091}P$ NCs recorded after 24-hour CP study, illustrating the [A] Co 2p, [B] P 2p, and [C] Mn 2p regions. The spectral data are represented by square symbols and colored lines are fitted deconvolutions.

Table S3. Comparison of HER overpotentials and Tafel slopes before and after 10 h of chronopotentiometry (CP) study in 1 M KOH.

Catalyst	Stage	Overpotentials (mV)			% increase of η			Tafel slopes	% increase of Tafel slopes
		η_{-10}	η_{-50}	η_{-100}	η_{-10}	η_{-50}	η_{-100}		
CoP	Pre-CP	166.70	315.78	434.92	16.13%	6.62%	4.71%	129.02	8.83%
	Post-CP	193.59	336.68	455.40				140.41	
$Co_{0.909}Mn_{0.091}P$	Pre-CP	136.29	186.53	214.91	5.83%	4.25%	3.29%	65.77	4.77%
	Post-CP	144.23	194.45	221.97				68.91	
Pt/C	Pre-CP	58.20	229.92	355.31	54.38%	5.58%	8.91%	62.31	37.79%
	Post-CP	89.85	242.76	386.97				85.86	

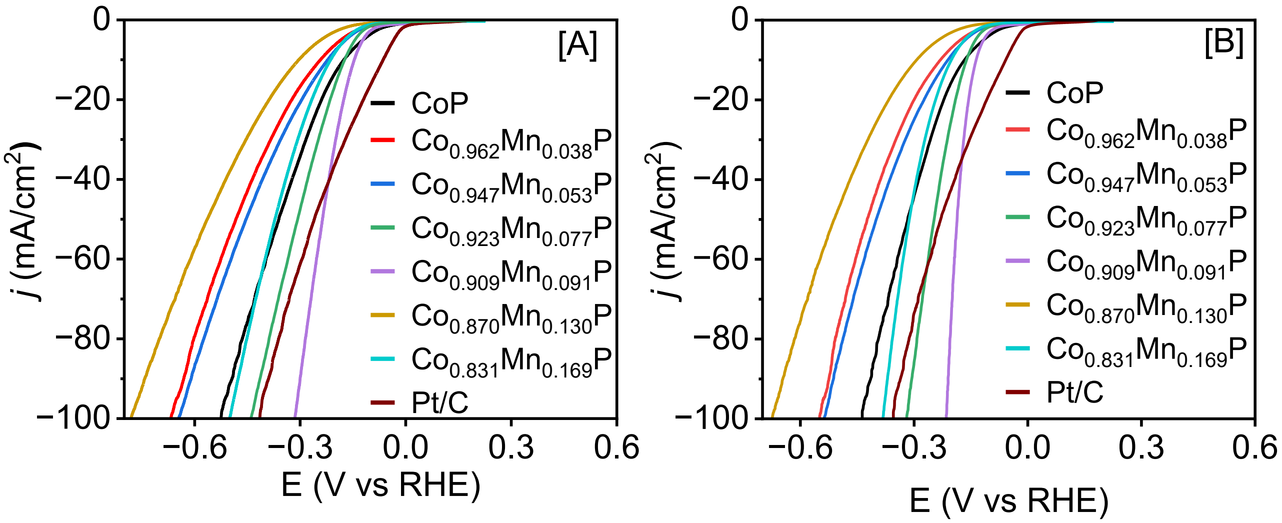

Figure S14. [A] LSV curves before *iR*-correction. [B] LSV curves after *iR*-correction using the sample-specific solution resistance values obtained from EIS Nyquist plots.

Table S4. Comparison of HER overpotentials before and after *iR*-correction for CoP, $Co_{1-x}Mn_xP$ NCs, and Pt/C catalysts in 1 M KOH.

Sample	Overpotentials (mV)							
	η_{-10}		η_{-20}		η_{-50}		η_{-100}	
	Before	After	Before	After	Before	After	Before	After
CoP	175.66	166.70	238.46	221.30	360.16	315.78	524.46	434.91
$Co_{0.962}Mn_{0.038}P$	242.06	230.44	322.96	299.79	480.86	422.84	668.76	547.34
$Co_{0.947}Mn_{0.053}P$	220.86	210.73	294.76	273.48	452.76	398.93	644.76	535.36
$Co_{0.923}Mn_{0.077}P$	172.66	161.65	214.76	190.77	305.06	247.10	438.26	319.78
$Co_{0.909}Mn_{0.091}P$	145.66	136.29	175.46	156.25	235.06	186.53	313.56	214.91
$Co_{0.870}Mn_{0.130}P$	302.96	292.96	387.86	366.31	562.96	509.16	780.26	671.41
$Co_{0.831}Mn_{0.169}P$	212.56	201.63	265.96	242.91	370.66	311.66	499.46	380.38
Pt/C	64.33	58.19	120.43	108.44	259.92	229.92	415.32	355.31

REFERENCES

(1) Lenin, R.; Joy, P. A. Role of Primary and Secondary Surfactant Layers on the Thermal Conductivity of Lauric Acid Coated Magnetite Nanofluids. *J. Phys. Chem. C* **2016**, *120*, 11640-11651.
